# BLANNOTATOR: enhanced homology-based function prediction of bacterial proteins

**DOI:** 10.1186/1471-2105-13-33

**Published:** 2012-02-15

**Authors:** Matti Kankainen, Teija Ojala, Liisa Holm

**Affiliations:** 1 Institute of Biotechnology, University of Helsinki, Helsinki, Finland; 2 Institute of Biomedicine, University of Helsinki, Helsinki, Finland; 3 Department of Biological and Environmental Sciences, University of Helsinki, Helsinki, Finland

## Abstract

**Background:**

Automated function prediction has played a central role in determining the biological functions of bacterial proteins. Typically, protein function annotation relies on homology, and function is inferred from other proteins with similar sequences. This approach has become popular in bacterial genomics because it is one of the few methods that is practical for large datasets and because it does not require additional functional genomics experiments. However, the existing solutions produce erroneous predictions in many cases, especially when query sequences have low levels of identity with the annotated source protein. This problem has created a pressing need for improvements in homology-based annotation.

**Results:**

We present an automated method for the functional annotation of bacterial protein sequences. Based on sequence similarity searches, BLANNOTATOR accurately annotates query sequences with one-line summary descriptions of protein function. It groups sequences identified by BLAST into subsets according to their annotation and bases its prediction on a set of sequences with consistent functional information. We show the results of BLANNOTATOR's performance in sets of bacterial proteins with known functions. We simulated the annotation process for 3090 SWISS-PROT proteins using a database in its state preceding the functional characterisation of the query protein. For this dataset, our method outperformed the five others that we tested, and the improved performance was maintained even in the absence of highly related sequence hits. We further demonstrate the value of our tool by analysing the putative proteome of *Lactobacillus crispatus *strain ST1.

**Conclusions:**

BLANNOTATOR is an accurate method for bacterial protein function prediction. It is practical for genome-scale data and does not require pre-existing sequence clustering; thus, this method suits the needs of bacterial genome and metagenome researchers. The method and a web-server are available at http://ekhidna.biocenter.helsinki.fi/poxo/blannotator/.

## Background

The rapid progress in sequencing technology has enabled the generation of unimaginable amounts of bacterial genomic data. The genome sequences of thousands of bacteria have been determined, and many more are in progress [1]. In addition, enormous numbers of sequences have been produced in metagenomic studies exploring the genomic contents of microbial communities by sequencing [2]. The interpretation of this data is necessarily based on computational analysis, and only a minority of the predicted protein-coding sequences are experimentally characterised or tested with functional genomics assays. Functional inferences for the large majority of putative proteins therefore require sophisticated and powerful annotation tools that can predict protein function based on sequence.

Many automated protein function prediction methods describe the biological role of a gene product in terms of single-line description of protein function (DE) or gene ontology (GO). These two widely used annotation schemes are radically different. The first shows a protein function in a human-readable free text format [3], whereas GO and its directed acyclic graph structure depict the function with a controlled vocabulary that is machine-readable [4]. Several valuable tools have been developed for the prediction of DE or GO annotations, but the most popular tools are based on the concept of homology [5-7]. The premise of this technique is that the functional properties of related sequences are conserved during evolution and that the function of the query protein can be inferred from that of other proteins with similar sequences. Among the many homology-based function prediction solutions [8-15], the simplest involves sequence search with tools like BLAST and PSI-BLAST [16] and the transmission of function between similar sequences [10,11]. Other variants include functional inference based on a set of related sequences, although pooling of information is often restricted to GO annotations [12-14]. In a typical genome, a large proportion of gene products can be functionally annotated by these kinds of methods [17]. Predictions are not however always accurate and estimates of the error rate of genome-scale annotation processes vary from 5 to 40% [5,6]. This approach is also vulnerable to misannotations, which were recently reported to exceed 80% for some protein families in some sequence databases [18]. Furthermore, transfer of correct functional annotations with these methods require high levels of sequence identity. It has been suggested that at least 40-60% identity is, for example, needed to accurately infer enzymatic function [5,6,19].

Two other forms of homology-based annotation use protein signatures and phylogenomics. In the first approach, the function is inferred from similarity to motifs and domains obtained from databases, including FIGfams, HAMAP or Pfam [20-22]. A greater coverage over the sequence space is achieved than with BLAST or PSI-BLAST, but these predictions may be overly general, and some resources report functional annotations on the domain rather than the protein level [5,6]. Phylogenomics directs the annotation process using the evolutionary history of sequences [5,6,23]. Commonly, function is inferred from that of orthologs, *i.e.*, sequences related by speciation, and less weight is given to paralogs that have evolved by gene duplication and may have acquired a new function [5,6]. For example, gene duplications can be inferred from incongruence between gene and species phylogenies [5,23]. Phylogenomics is ideal to identify the most likely function out of many candidates associated with a set of protein sequences that share sequence similarity with the query protein. However, current implementations are often inefficient for genome-scale analysis, and some methods require a predefined species tree, which is not often at hand when analysing bacterial data.

An attractive alternative to the phylogenomic construction of sequence sets is to cluster sequences according to their annotation similarity. This approach has the advantage that the necessary information can be extracted from public data repositories, and time-consuming all-against-all protein comparisons to generate phylogenetic trees or orthologous sequence sets are not required. The use of annotation similarity has been shown to be beneficial for processing protein function [12,24]. For example, CLAN combined sequence- and annotation-based clustering and successfully identified inconsistently annotated proteins from SWISS-PROT [24]. On the other hand, in ConFunc, sequences identified by PSI-BLAST are split into groups according to their GO terms, and these sequence groups are used to build up feature-derived sequence profiles from which protein function can be predicted [12]. GO annotations at high levels of precision were created, even at levels of sequence identity equal to or below 30% [12].

We present a computational method for protein function prediction that relies on the concept of homology to annotate a query sequence with one-line summary descriptions of protein function. This tool, called BLANNOTATOR, splits sequences identified by BLAST into groups according to their DE and GO annotations. The information from database matches that belong to same functional group is then pooled to evaluate DEs. The general scoring scheme we use follows that used in an earlier solution [8]. The idea underlying BLANNOTATOR is that a correctly annotated sequence will occur in the list returned by sequence comparison, but its position(s) in the list may be arbitrary. For example, some protein sequences detected in the search may be related but un- or misannotated, and others may not be related at all. If misleading subject sequences have populated the top of a match list, conventional function prediction methods may fail to recognise the best annotation. In contrast, our method circumvents this problem by building the DE evaluation upon multiple sequence matches with common functional annotations. The concept of annotation-guided sequence clustering borrows from CLAN and ConFunc [24,12], but unlike these programs, our method assigns sequence similarity search results to groups based on two types of functional information. This produces more comprehensive clusters because the two annotation schemes can complement each other's defects; in the absence of GO annotation, links can be formed via DEs, and misspelled or erroneous DEs can be rescued using GO-based links.

The performance of BLANNOTATOR was assessed by predicting protein functions for a set of 3090 bacterial proteins available in the SWISS-PROT database [3]. While building the evaluation data, we aimed to minimise the number of circularly referenced functional annotations among the test sequences and BLAST hit lists. Accordingly, we discarded hits in which the query sequence matched a sequence that had a newer submission date than the annotation date recorded for the query protein. The DE of the remaining sequence hits was returned to its previous state (*i.e*., prior to the annotation date) with the help of UniSave [25]. The proportion of character differences between the predicted and correct annotation was then computed to provide an estimate of the annotation quality. Using this data, predictions made by BLANNOTATOR were at minimum 1.6-fold better than those of the five other tested methods. Homology-based transfer of function can be ineffective in the absence of high levels of sequence identity. To simulate this scenario, the SWISS-PROT data were reanalysed after the removal of sequences with greater than 50% identity to the query sequence. The conclusions drawn at this limited level of sequence identity were for the most part consistent, showing that our method also performs well under these restricted conditions and is better than the other homology-based transfer methods we tested.

We analysed the protein-coding sequences of *L. crispatus *strain ST1 [26] to further demonstrate the functionality of BLANNOTATOR. Human inspection of the resulting function predictions showed that our method provided a valid annotation for ~85% of the originally characterised cases. The original characterisation was based on a manual review of the results of a compendium of bioinformatics approaches [26]. In comparison, RAST- or top BLAST hit-based approaches provided a valid function prediction for ~58% and ~69%. Moreover, BLANNOTATOR's ability to structure BLAST data in clusters and its user-friendly output were found to facilitate the annotation process.

## Results

**Figure 1:**
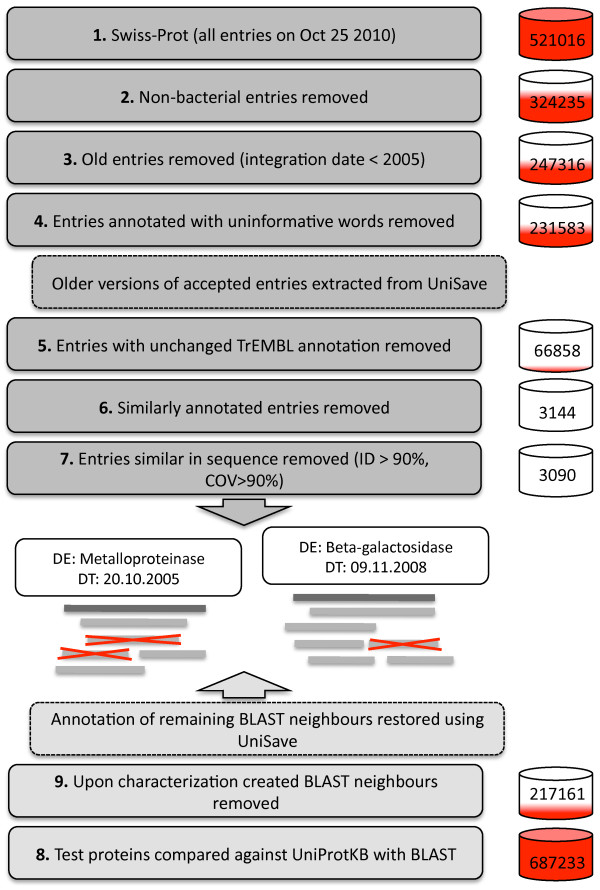
**Schematic representation of the construction of the SWISS-PROT dataset used to assess the performance of automated protein function assignment methods**. Test proteins (dark grey boxes) were initially selected by extraction from the entire SWISS-PROT database. The extraction protocol involved the removal of non-bacterial entries, the removal of entries created ahead of 2005, the removal of entries with words '*UPF*' or '*uncharacterized*', the selection of entries added directly to SWISS-PROT or that had undergone revision since their storage in TrEMBL, the removal of similarly annotated entries and the removal of entries showing sequence similarity to each other. The construction of the sequence similarity search results (light grey boxes) for functional inference included a sequence comparison against UniProt with BLAST, the removal of BLAST hits to sequences for which the creation date was newer or equal to than the annotation date of the query sequence and the restoration of the annotations of the remaining BLAST hits to their status just before the annotation date. Barrels show the number of entries and BLAST hits that passed each filtering step, and the intensity of the red colour indicates the corresponding fractions. White boxes in the crossing area show the annotation (DE) and the annotation date (DT) for two test sequences. Red crosses indicate BLAST hits that were removed.

To assess the success of protein function prediction, function was predicted with BLANNOTATOR and five other methods for a set of bacterial proteins of known function retrieved from SWISS-PROT. This evaluation dataset was constructed by extracting bacterial protein sequences that had been deposited in the database after 2005 and either had been added to the database directly or had been associated with a substantially different DE while stored in the TrEMBL section. To maximise the diversity of the test dataset, a random representative was selected for every different DE present in the initial data, and the first appearance of the present database annotation of the sequence (annotation date) was recorded. A total of 3090 protein sequence entries out of 327174 candidates fulfilled our search criteria. Sequences were compared against the entire UniProt database using BLAST, resulting in approximately 690,000 significant matches. To minimise the number of circularly referenced annotations among targets, we discarded hits to sequences with a creation date newer than the annotation date of the query protein. This affected ~68% of the initial matches, and the final data thus consisted of ~220,000 matches. In total, 58 of the 3090 test sequences did not show similarity to any other sequences, and the remaining majority retrieved an average of 72 protein sequences each. To complete the removal of circular references, the remaining DEs were returned to their states prior to the annotation of the query using UniSave [25]. The steps involved in the construction of the evaluation data are depicted in Figure 1.

### Characterisation of the evaluation data

**Figure 2:**
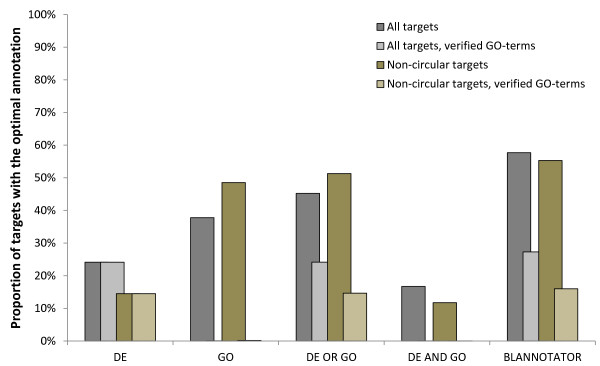
**The proportion of correctly annotated BLAST hits in the SWISS-PROT dataset**. The mean of proportions of BLAST hits with function descriptions similar to those of their query sequences was recorded before (grey bars) and after (brown bars) the removal of circular referencing. Statistics was computed using all GO terms (darker bars) and by accepting only GO terms with experimental or computational evidence codes (lighter bars). BLAST hits were selected based on DE annotation, GO annotation, GO or DE annotation, GO and DE annotation, and BLANNOTATOR. The reported means were calculated over all test protein sequences.

To study the complexity of the function prediction process, we computed the proportion of BLAST hits that had an annotation in common with its query sequence. We called such annotations optimal because their transfer would also mean the transfer of a correct function to the query. Here, an optimal DE was required to have a modified Levenshtein distance (mLD) of 0.00 compared to the actual database annotation of the query. The mLD metric is based on the Levenshtein distance (LD) [27] and gives the fraction of character changes between two annotations. Differing from LD, our distance metric does not however constrain the word order. Thus, this metric gives the best possible score of 0.00 for annotations like '*DNA gyrase A subunit*' and '*subunit A DNA gyrase*'. An optimal GO annotation of a subject sequence was required to have the same set of GO terms that was associated with its query. GO annotations were based on data extracted from GOA [28]. Figure 2 shows the mean of percentages of BLAST hits with optimal annotations. On average, 15% of matches had the optimal DE. However, the proportion varied considerably: for 60 test sequences, over 90% of BLAST hits were associated with the optimal DE, whereas 1903 other test sequences had no optimally annotated hits in their match lists. This also informs our picture about the level of difficulty of the function prediction process. Finding the correct DE for the first set is trivial, as the correct function can be inferred from almost any BLAST hit, whereas the characterisation of the latter set is challenging due to the absence of optimal annotations, even though we consider partial word matches as partially successful in our later analyses. For comparison, Figure 2 shows the statistics before and after we minimised circular referencing and returned the BLAST hit to its previous state. The figure also shows the effect of accepting only verified GO terms. In comparison to DE statistics, the optimal GO annotation was on average three-fold more often associated with the BLAST hits of a test sequence. This is likely due to the structured format and high level of uniformity of GO annotations. Taking a union of the BLAST hits with the optimal DE or GO annotation, a slightly larger set was obtained than when selection was based on only optimal GO annotations, indicating that the set of BLAST hits with the optimal DE and the set with the optimal GO overlapped significantly but also contained different elements. The largest set of database hits was extracted using our strategy (see Figure 2). This set included non-optimally annotated BLAST hits that were considered valid by our method. For example, all sequence hits associated with a DE synonymous to the optimal DE were included in a set of optimally annotated hits, if one of these synonymously annotated hits was associated with the optimal GO annotation, providing an intermediate hit with which to rescue other database matches with the synonymous annotations.

### Effects of sequence filtering

**Figure 3:**
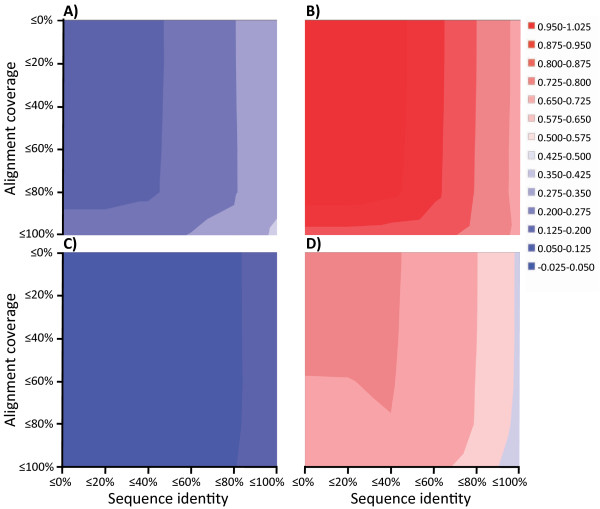
**Mean annotation quality of the ideal and worst-case predictions in SWISS-PROT dataset**. Modified Levenshtein distance-based statistics is shown for the ideal (panels A and C) and worst-case (panels B and D) predictions after removing BLAST hits at various sequence identity and alignment coverage thresholds. Panels A and B show the statistics for a dataset from which circularly referenced annotations had been removed, and panels C and D show the statistics for a dataset in the presence of circularly referenced protein annotations. Red colours indicate bad predictions and blue colours good predictions.

To establish the theoretical limits of homology-based function prediction in our data, the minimum, median and maximum mLD were recorded for each test sequence, and the means and standard deviations were calculated. This analysis was repeated several times after the removal of sequence hits below a given identity and alignment coverage to the query sequence (see Figure 3 and Table S1 of Additional file 1). In total, ~97% of test sequences had at least one recorded BLAST hit in the absence of sequence filtering, setting the upper limit on the number of cases for which homology-based transfer can assign a function. With these settings, the means of the minimum (the ideal function prediction achievable) and the maximum (the worst-case prediction) mLD were 0.23 and 0.94, respectively. In contrast, only ~6% of the test sequences had a match that fulfilled the criteria of perfect sequence identity and alignment coverage. The use of 100% identity and alignment coverage also affected the measured annotation quality parameters, and a higher mean of the minimum mLD and lower mean of the maximum mLD were recorded. The change in annotation quality was not, however, restricted only to this strictest threshold combination. In general, the removal of BLAST hits with increasing identity threshold produced throughout the data more suitable worst-case predictions. The benefit of the improved worst-case predictions was diminished by the increasing mean quality of the ideal predictions. Particularly substantial numbers of suitable DEs vanished at the 60% identity threshold (see Figure 3). On the other hand, only a few alignment coverage thresholds were noted to have a severe impact on the annotation quality. The annotation coverage and quality were substantially influenced only when the alignment had to cover the entire subject sequence. Furthermore, a similar trend was observed when BLAST results were filtered with relative bit score, meaning that the use of stricter parameters lead to more successful worst-case predictions, but yield less optimal best-case predictions. In here, bit score was compared to the query's maximal self-BLAST bit score (see Figure S1 of Additional file 1). In addition to above analysis, the effect of sequence filtering was studied before the regression operation. These results were mainly consistent with those described above (see Figure 3, Figure S2 and Table S3 of Additional file 1). Differences included a greater spread of median mLD values (from 0.31 to 0.40), which is more than double the spread that was seen after correction, in which case the values ranged from 0.49 to 0.53. This means that improved predictions were possible with the full dataset and indicates that the median annotation quality relies only little on the chosen parameterisation. Secondly, the difference in quality between the extreme predictions was generally smaller for a particular parameter setting. For example, focusing on analyses based on hits with sequence identity ≥ 40%, the mean annotation qualities of the ideal and worst-case predictions in the presence of circularly referenced protein annotations were 0.17 and 0.66, respectively, whereas the corresponding values obtained after the removal of circular referencing were 0.25 and 0.90, respectively (see Tables S1 and S2 of Additional file 1). Tables S3 and S4 show the results of the above analyses after removing sequence hits with greater than 50% identity to the query sequence, both before and after circularly referenced BLAST hits had been removed (see Additional file 1). These results are consistent with those described above.

### Performance evaluation

**Figure 4:**
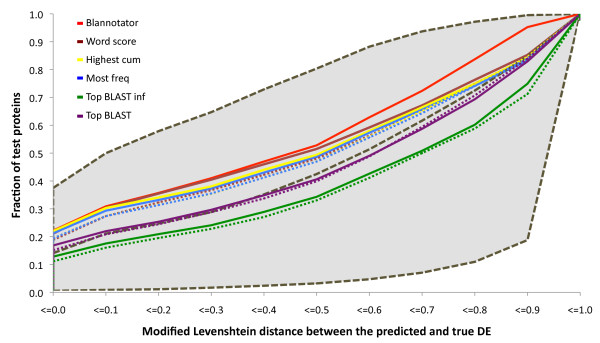
**The quality of automated protein function predictions**. The fraction of predictions below a certain modified Levenshtein distance from the correct annotation is shown. Function prediction was performed from an unfiltered BLAST match list and a list from which circularly referenced annotations had been removed. Function prediction was based on the most significant BLAST match (purple), the top BLAST match without any uninformative words (green), the most common annotation among BLAST hits (blues), the annotation associated with the highest bit score sum (yellow), a word-based scoring scheme (brown) and BLANNOTATOR (red). Dashed lines show the performance of the tool when applied to the largest group of matches sharing a common GO annotation. The black dashed lines and the grey background indicates the theoretical level of performance when the ideal, median or worst-case predictions were chosen.

The success of the automated protein function prediction was assessed by measuring the similarity of the predicted and correct DEs in terms of mLD. Partial word matches were considered partially successful. In addition to BLANNOTATOR, five other methods were used to assign functions. Two of these relied on a single annotation inferred from the functional description of *i*) the most significant sequence identified by BLAST or *ii*) the most significant sequence identified by BLAST that had a functional description without any uninformative words. Function annotations were also inferred based on *iii*) their frequency in the BLAST hit list, *iv*) the sum of bit scores of the associated matches and *v*) the highest word-based score. The annotation processes of the five other solutions were also done by restricting analyses only to the largest group of sequences that shared a common GO annotation. The quality of the function prediction process and the fraction of predictions with an mLD below a given threshold is shown in Figure 4. Four methods generated the optimal DE for approximately 20% of the proteins, and nearly half of the predictions had an mLD below 0.50. The common denominator between these four methods was that they all based their predictions on information from multiple BLAST hits. The highest fractions of DEs in all bins were obtained with BLANNOTATOR. In the first six bins, the improvement was borderline significant, but our method stood out in the remaining bins. It surpassed the second best method by 109 predictions in the seventh bin (mLD ≤ 0.60), and even greater levels of improvement were recorded thereon (see Figure 4). On the other hand, inference of protein function from a single sequence was clearly a less successful strategy. The annotation quality of such predictions was, in most cases, not any better than the expected median annotation quality of the prediction. Another view of these data is provided in Tables S5 and S6, which show the mean and standard deviation mLD statistics and the standard score (Z-scores) mLD statistics for each method (see Additional file 1). The values in the above tables were calculated at different identity and alignment coverage thresholds to test the robustness of functional inference with respect to sequence filtering. Under all filtering settings, the Z-score received for BLANNOTATOR was at least 1.6 times better than that of the second best method. Other conclusions drawn from these data are the same as those drawn from Figure 4. We also performed function prediction and calculated the subsequent statistics from the original BLAST data prior to the removal of circularly referenced annotations, in the absence of sequence hits with greater than 50% sequence identity with the query and with a combination of both criteria (see Figures S3-S5 and Tables S7-S12 of Additional file 1). The results of these analyses are consistent with the others described here. In some analyses, *e.g*., the one after the removal of sequences above 50% identity, even greater success was recorded for our method.

### Annotation of protein-coding sequences of *Lactobacillus crispatus *strain ST1

The functionality of BLANNOTATOR was also tested by assigning annotations to the protein-coding sequences of *L. crispatus *strain ST1 [EMBL: FN692037] [26]. In this example, function prediction was based on BLAST data from which sequence hits with lower than 40% identity and 50% alignment coverage to the query were discarded to remove spurious hits. Our method predicted functions for 1711 of the 2024 protein-coding sequences analysed. To assess the validity of the reported annotations, a human operator (TO) reviewed the predictions. This analysis revealed that our method made a prediction that matched the original for ~85% (1377) of the cases with prior functional annotations. In the remaining cases, the assigned annotation was either biologically distinct or less specific. In addition to our method, we performed function predictions with RAST [20] and by inference from the top BLAST hit. These two methods generated annotations for 1071 and 1498 test sequences, covering ~53% and ~74% of the proteome. Evaluation of the predictions of the methods showed that RAST and the top hit from BLAST generated fewer acceptable functions than our method. They produced the supported annotation for ~58% and ~69% of the sequences with approved functional annotation, respectively, and defined valid annotations for 104 and 103 protein-coding sequences that our method had failed to assign the correct function, while our method produced acceptable annotations for 530 and 362 protein-coding sequences for which the RAST- or BLAST-based annotation was unsuccessful.

### Selected examples from the annotation results

Examples of selected protein-coding sequences for which our method assigned the human curator-supported function are given below.

**• Multiple and diverse candidate annotations**. LCRIS_00064 is an example of a sequence that only BLANNOTATOR managed to characterise similarly to InterProScan [29]. The competing methods failed to assign a function to this sequence, most likely because of the related sequences of LCRIS_00064 were associated with seemingly incoherent functional annotations, and the top candidates did not have valid annotations. For example, matching sequences were described with a set of annotations ranging from '*50S ribosomal protein L3*' and '*Uracil-DNA glycosylase*' to the correct annotation, '*Cell wall-associated hydrolase*'. Moreover, the correct functional description was enriched in the less conserved related sequences. Out of the 40 top hits, only seven had the valid functional description, while a third of the accepted hits in total had been described with this particular annotation.

**• Inference of specific enzymatic activity**. An example in which an enzymatic function was recovered by BLANNOTATOR is that of LCRIS_00495. This putative protein had acceptable sequence level similarity to ten sequences, most of which had no functional information. In spite of the limited amount of data that could be used in homology-based transfer, the query protein was defined as an '*Antibiotic biosynthesis monooxygenase*' by BLANNOTATOR, similarly to InterProScan [29]. The other methods generated DEs that were less informative: '*Putative uncharacterized protein*' and '*putative phosphoglycerate mutase*'. Our method also defined an enzymatic function for LCRIS_01619, which was identified to be '*1,4-dihydroxy-2-naphthoate octaprenyltransferase*', similar to the function that was obtained using COG [30]. The two other methods failed to assign a function to this putative protein.

**• Transferring meaningful annotations from remote relatives**. Only BLANNOTATOR predicted a function for each component of the clustered regularly interspaced short palindromic repeats (CRISPR) system, whereas the competing methods failed to infer functions for LCRIS_01207 and LCRIS_01212, both of which are components of this vital defence mechanism that provides acquired phage resistance in bacteria and archaea [31]. LCRIS_01207 and LCRIS_01212 showed only remote similarity to the CRISPR-associated protein sequences in the database, and BLAST hits associated with meaningful functional information had limited (≤ 50%) identity to the queried protein. For example, LCRIS_01207 had acceptable similarity to 59 sequences annotated as '*CRISPR-associated protein, Cas2*', but these sequences displayed a maximum identity of 45%. Thus, if annotations had been predicted from top matches or the analysis of remotely similar sequences had been bypassed, no function could have been assigned to these sequences, leading to a failure to characterise the CRISPR system.

## Discussion

**Figure 5:**
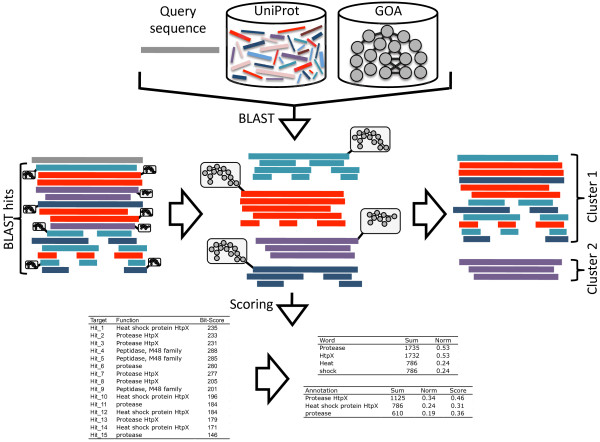
**Outline of the BLANNOTATOR method**. Related sequences (coloured bars) are detected using BLAST against UniProt, GO annotation information (grey circles) is extracted from GOA, sequence hits are organised into groups according to their DE and GO annotation and DE annotations are scored. In the example shown, 15 BLAST hits, described by four DE annotations (red, cyan, violet and dark blue bars) and two GO annotations (the grey circle diagrams), are split into two clusters. Initial BLAST bit scores, as well the final and intermediate scores, are shown for the larger of the two clusters.

Here, we present a computational method that can infer the biological role of a protein based on its sequence. BLANNOTATOR builds upon the concept of homology to annotate bacterial protein sequences in terms of one-line summary descriptions of protein function. In contrast to most other annotation methods based on the same strategy, the function prediction process is performed based on sets of sequences that are free of functional discrepancies. Conflicting functional annotations between sequences identified by BLAST are resolved in a two-step procedure in which matched sequences are split into groups according to their functional annotations. In the first step, matches that have the same DE are grouped. Then, more linkages are formed between sequence hits that are associated with the same set of GO terms, followed by the selection of the candidate functional annotation that has the most support in the BLAST match list (see Figure 5). In our test data comprised of bacterial SWISS-PROT entries, this annotation-guided clustering extracted on average ~55% of the database hits of the query. This was the largest pool of database hits and contained ~6% more hits than the second biggest set of sequences that was discovered with the combination of GO or DE annotations (see Figure 2). This large proportion of sequences was retrieved because our method was able to join pairs of differently annotated hits together when it found one intermediate hit that shared one similar annotation with both of those hits. For example, a set of sequence hits with synonymous, but non-optimal DEs are rescued if even one has the optimal GO annotation. In this case, this sequence with the optimal GO annotation provides an intermediate by which all other sequence hits with the same non-optimal DE annotation can be joined with those having the optimal GO annotation. The ability of our method to select more database matches is likely related to the propagation of erroneous annotations and varies across datasets, but, in principle, our method seems to be able to coup with heterogeneous annotations, a factor that often complicates the annotation process. It is also worth noticing that exclusion of unverified GO terms did not paralyse our method. On average, below 1% of the BLAST hits had same verified GO terms as the query had, making this subset of database hits too tiny for practical functional inference. In contrast, ~16% of targets were discovered using our method under the same circumstances: this was about a third of the value obtained without the restriction, but most likely still sufficient enough to predict a valid annotation for a test sequence, and slightly (a bit over 1%) better than that of the second best approach (see Figure 2). This result thus suggests that our method can achieve good performance using only verified GO terms, but also indicates that use of all GO terms is recommendable.

The idea underlying BLANNOTATOR is that the reorganisation of sequence similarity search results can improve homology-based protein function prediction. For example, sequence similarity search tools may find undesired sequences, and the match list may not always be in the ideal order [5]. Commonly, sequence grouping in protein function analysis is performed through the identification of orthologs, which can be performed using any of the many algorithms designed for that purpose [32]. Similar to the principal aim of forming orthologous sequence sets in automated protein function prediction, our annotation-guided clustering and simple multiclass classifier produces sets of proteins that are likely to have common functions. However, our program does this without dealing with complicated phylogenies or requiring time-consuming all-against-all sequence comparisons. Likewise, in our method, database hits associated with differing functional descriptions are not assigned to the same group, which effectively mimics the discarding of paralogous and other non-conserved sequences that occur in phylogenomic techniques [33-35]. Moreover, it was recently reported that sequence similarity could be better than sequence orthology in terms of predicting protein function [36,37]. Thus, functional inference from orthologous sequence sets may be overkill and may even reduce the quality of function predictions in addition to increasing processing time. In comparison, BLANNOTATOR circumvents this deficiency as its function prediction is based on sets of sequence hits generated using functional information and because the candidate annotation is selected by pooling information over multiple hits in a sequence set.

We scored the DE of sequences identified by BLAST with a method that is based on an earlier proposal [8]. The basic principle of this scoring scheme is that higher scores reflect higher sums of bit scores for the database matches to a given annotation. The scoring scheme we used assumes that each target sequence provides an additional layer of support and is independent of the other matches. These assumptions are suitable for protein function prediction in most scenarios but fail if the identified sequences have a biased functional representation. For example, if a single researcher has identified his/her favourite protein from many closely related organisms and annotated each protein similarly, bias will be introduced to the results, and the significance of the over-studied annotation can be over-estimated. Some computational approaches tackle this kind of bias. For example, ConFunc uses all-against-all sequence comparisons to reduce bias [12], and CLAN pre-calculates the annotation frequencies in the database to address the error [24]. However, here we have traded the minor loss in quality for a greater gain in performance. Also, this problem can partially be circumvented by the use of a less redundant sequence database, such as UniRef90 or UniRef50 [38].

In order to assess protein function prediction methods, a well-established test data is required. The ideal evaluation data should contain only proteins and protein functions that have been characterised experimentally. This data should also list all of the alternative functions of the protein, irrespective of the conditions under which the function is active, because a predicted function can confidently be declared erroneous, or correct, only if all of a protein's true functions are known. Moreover, test sequences should include proteins with a variety of functions and varying levels of similarity to other sequences. Finally, this rich source of functional information should not be propagated further to avoid circular referencing. Here, we first evaluated function prediction by characterising a set of bacterial proteins of known function extracted from SWISS-PROT [3]. Our data is not perfect but has several positive aspects. First, function annotations have passed the strictest filter possible and are as complete as they can be because SWISS-PROT has the lowest annotation error level of all of the major sequence databases [18]. Test sequences displayed a wide variety of functions, both enzymatic and non-enzymatic, and they showed similarity to a varying range of other sequences. Moreover, functions for these test sequences were inferred from data that had a minimal number of circularly referenced annotations (see Figure 1). However, our evaluation dataset has its own problems. For example, we could not exclude the possibility that UniProt was not used for function prediction in the first place. If this was the case, there could be an increased chance that the same annotation would be produced again. Functional annotations can also be wrong, incomplete or contain mistakes, although they were selected from the most reliable database. Furthermore, there was no obvious way to regress GO annotations, so newer sets of GO terms had to be used in the annotation process. In addition to our novel test data, a new machine-based strategy was developed for DE comparison and for judging if two annotations are the same. This metric, mLD, computes the fraction of character changes between two annotations and can deal with word order changes. However, similar to most string metrics, it cannot process adequately synonymous terms. Annotations like '*GyrA*' and '*DNA gyrase, subunit A*' are falsely considered to be different, while erroneous similarity is seen between '*DNA polymerase I*' and '*DNA polymerase IV*', if these annotations occur in the same match list. Additionally, the method is blind to annotations of differing specificity. For example, '*DNA polymerase' *is a good match with the two previous matches.

The annotation performance of BLANNOTATOR and the five other methods in the SWISS-PROT data was assessed using mLD statistics. At first we computed the fraction of predictions that were equal to or smaller than a certain distance from the valid database annotation of the query (see Figure 4). We chose to use this approach instead of the more typical sensitivity and specificity analysis, because it is difficult to draw the line between acceptable and non-acceptable annotations. Is an annotation correct, if it differs from the valid by, for example, three characters? The results shown in Figure 4 indicate that our method was better than the other function-assigning methods tested. In total, 22% of all predictions made by our method matched the one given to the query, and over 50% of the predicted annotations had an mLD equal to or smaller than 0.50. When circularly referenced functions had not been removed, even better performance was observed. The optimal DE was predicted for 37% cases and an mLD of 0.20 or lower was recorded for over half of the predictions in this data (see Figure S4 of Additional file 1). The standardised mLDs also supported the improvement and showed that predictions made by our method were, on average, 0.13-0.43-fold (after removal of circularly referenced annotations) and 0.10-0.46-fold (before the restoration process) better than the expected mean quality of the prediction. In contrast, the second best method improved results by 0.00-0.23-fold, and methods that relied on a single match, often the first method used in genome projects, were worse than a random selection from the BLAST hit list (see Table S5 of Additional file 1). We believe that the success of our method in protein function prediction is a sum of the whole process - the filtering of unspecific and infrequent words, the solving of functional conflicts and the use of the scoring system - and that each step contributes to the outcome. The use of new GO terms might have altered the results slightly, *i.e*., our method might not have generated the same partitioning as it would have generated on the date that the test protein was annotated. This effect most likely is marginal, if it exists at all, especially because we recorded the quality of the DE and not of the GO annotation. However, to rule out the possibility that the use of new GO terms has given some extra favour to our method, the other methods were also tested by providing them with only the information from the largest sequence hit group (if consisting of more than five matches) sharing a common GO annotation. The performance of the other methods was generally worse than the analysis made without this restriction (see Figure 4), suggesting that no extra benefit was given to our method.

In a genome project, automated protein function prediction is often performed after the removal of dubiously related database matches. This is most commonly achieved by discarding sequence hits below a given sequence identity or alignment coverage to the query sequences. In Figure 3 we depict the effect of sequence filtering on annotation quality in the case of SWISS-PROT data. Contrary to earlier suggestions [4,5,19], our data encourage the use of modest identity or alignment coverage filters in function assignment because aggressive thresholds just result in fewer matches, irrespective of their suitability for function prediction. In these data, the highest thresholds yielded the poorest predictions. Requiring perfect identity and alignment coverage, the mean annotation quality of the best-case prediction was approximately 50% worse than in the absence of sequence filtering. A drop of approximately 25% was seen at the 60% identity threshold, indicating that even this threshold may be too high. Higher thresholds for alignment coverage also decreased the performance but had smaller effects than the identity parameter. Only the strictest (100% coverage) filter needs to be avoided. Statistics obtained from the data before it was regressed showed a similar, albeit weaker, trend (see Figure 3). However, under this condition, the mean of the median mLD fluctuated more and was over twice (0.09) the value that was seen after the regression operation (0.04). Our data thus indicate that if BLAST hits must be removed, it should be done with modest sequence identity (0-40%) and alignment coverage (0-80%) filters. Results of these analyses were consistent with those in which BLAST results were filtered using relative bit scores, indicating that the same tendencies applies to that parameter also (see Figures S1 and S2 of Additional file 1). Our findings thus suggest that rather than discarding matching sequences, more focus should be placed on finding the true signal from the complete list of database matches. For example, weak signals could be amplified by analysing sequence sets, which is the main principle of BLANNOTATOR.

In addition to the SWISS-PROT data described above, we assessed our method by analysing the proteome of *L. crispatus *strain ST1. As the genome was unpublished at the time of analysis [26], the risk of circular referencing was again abolished. This more realistic setting may also have contained sequencing or gene prediction errors, which are likely to occur in modern genome projects. Moreover, the test set included a set of orphan protein-coding sequences that are specific to this particular organism, as well as more universal bacterial proteins that are required for a bacterium to live and operate. Also, to compensate these defects of mLD, prediction performance in this data was assessed by a human curator. This strategy avoids many of the problems described above, such as the recognition of different levels of specificity and synonymous wordings, but can produce other types of mistakes that are specific to human evaluation. The analysis of the putative proteome of *L. crispatus *strain ST1 [26] showed that our method performed well also in this data and inferred the preferred functional description for 85% of the originally characterised test sequences. The two other methods tested were less successful. Moreover, these and a set of other methods used in the original analyses had a lower annotation coverage of the putative proteome: the fraction of annotated sequences ranged from 80 to 3% for InterProScan, inference of function from the top BLAST match, COG, RAST, KAAS and HAMAP-scan, in order from best to worst [29,30,20,39,21]. In fact, our method had greater annotation coverage (85%) than even InterProScan, which is considered one of the most comprehensive and heavily used resources [5]. This can be explained by the effective use of all data. For some protein sequences, like that of LCRIS_01207, the preferred function was inferable only from matches showing low identity to the query. However, when working with sequences with limited levels of identity, the risk of inferring an erroneous function becomes higher, which is why functional information needs to be pooled.

## Conclusion

The large number of sequences provided by bacterial genomic projects has enabled the identification of an increasing number of bacterial protein-coding sequences. As only a limited fraction of these newly discovered putative proteins undergo an experimental characterisation, there is a need for sophisticated annotation tools that can infer the biological role of a protein based on its sequence. We describe a computational method for the automated prediction of bacterial protein functions. This tool, BLANNOTATOR, generates accurate one-line summary descriptions of protein functions. Our method is better than other methods that we tested and produces an output that is easily understood. Moreover, it is applicable to genome-scale datasets and can be used to analyse metagenomics data because it does not require phylogenetic information or additional functional genomics experiments. We believe that BLANNOTATOR will be very useful for discovering information about genome and metagenome sequences in the future.

## Methods

The BLANNOTATOR method is outlined in Figure 5. The function prediction process described here is based on the default settings. Sequence similarity search is performed using BLAST against UniProt [16,3]. GO terms are extracted from GOA [28], and GO annotations are created by pooling GO terms over each of the three ontologies and over their ancestor GO terms until the ontological root is reached. By default, all GO terms are accepted irrespective of their evidence type, but the user may choose to use only GO term with certain evidence types. GO annotations containing fewer than three different GO terms are discarded. This effectively removes uninformative annotations consisted of a few GO terms, which lie close to the ontological root, like the ' *cellular process*'. DEs, originating from the header section of the FASTA-formatted sequence, are obtained from the BLAST hit list. Uninformative and infrequent words are removed from the DEs. The list of uninformative words contains 21 terms noted to occur frequently in protein annotations: '*hypothetical*', '*uncharacterized*', '*putative*', '*contig*', '*predicted*', '*probable*', 'f*ragment*', '*genome*', '*protein*', '*chromosome*', '*possible*', '*similar to*', '*proteins*', '*homolog*', '*possible*', '*conserved*', '*homologous*', '*complete*', '*shotgun*', '*cdna*', '*family*'. In addition, infrequent words are removed from DEs that also contain frequent words. An infrequent word is defined as a word that occurs only once in the DEs of ten or more BLAST hits. If the test sequence has less than ten matches, infrequent filtering is not applied. If the DE processing creates an empty annotation, the hit is removed from the BLAST match list.

The remaining database hits in the BLAST match list are grouped according to their DE and GO annotations. At first, the annotation-guided clustering creates groups based on the DE information. Database hits are linked if they have the same DE. Hits or sets of hits identified in the previous step (an element) are then linked based on their associated GO annotations. Two elements are linked if their top-scoring database matches with accepted GO annotations (*i.e*. the sequence hit that has the highest bit score and has at least three GO terms) have identical GO annotations. Alternatively, GO annotations of several hits can be used to form links. In this case, hits within an element are ranked in descending order by their bit score and the GO annotations of every *n*th hit, read from the top, are merged and used in the comparison. The step of this interval is user-definable. Again, element joining requires identical GO term sets. Pooling of GO terms increases the run time substantially. Here, only the topmost GO annotations were used.

### Annotation scoring scheme

The DE scoring employed in BLANNOTATOR is similar to an earlier method [8]. In this scoring scheme, the DE is deconstructed into individual words. Each word is scored and normalised against the sum of bit scores of all BLAST hits for the query sequence. In brief, the score of word *i *associated with a sequence hit belonging to a group *j *in a set of *n *matches is

$$w_{ij}=\overset{n}{\underset{m\in S\left(i,j\right)}{\sum }}B_{m}/\overset{n}{\underset{l=1}{\sum }}B_{l}$$

where *S*(*i, j*) is a set of sequence hits in the group *j *that contain the word *i *in its DE and *B *is the bit score of a BLAST hit. A similar score is calculated for each DE *k*. This score is calculated by taking a sum of the bit scores of BLAST hits described with *k *and dividing it by the sum of bit scores of all of the BLAST hits for the query sequence

$$S_{k}=\overset{n}{\underset{m\in k}{\sum }}B_{m}/\overset{n}{\underset{l=1}{\sum }}B_{l}.$$

By normalising scores against the sum of the bit scores of all of the BLAST hits for the sequence, instead of normalising it against the sum of bit scores of all of the BLAST hits within the group, we ensure that DEs in different groups are unlikely to return equal scores. The final score of a DE is obtained by taking an average of the word and sentence scores of the DE.

### Implementation of BLANNOTATOR

**Figure 6:**
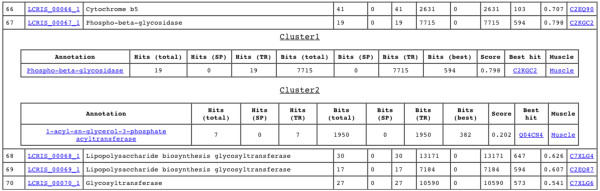
**A screenshot from the BLANNOTATOR web server showing the results page**. The BLAST hits to the protein sequence LCRIS_00067 were assigned to two groups, reflecting the fact that matching proteins are involved in two molecular functions. The results indicate that the protein could be described as either '*Phospho-beta-glycosidase*' or as '*1-acyl-sn-glycerol-3-phosphate acyltransferase*'. The first function describes proteins that have a hydrolase activity and act on glycosyl bonds, whereas the second function describes proteins that have transferase activity and transfer acyl groups other than amino-acyl groups. Our tool suggests that the protein of interest is more likely to have the hydrolase activity.

BLANNOTATOR is available for use on the program website [40]. These pages also include a web interface for the analysis of small test sets. The web-server takes a list of FASTA formatted sequences as the input and outputs functional descriptions of protein function. The user can specify a number of parameters in the web-server. Some of these can be used to remove sequence matches that do not match the query sequence well enough, including parameters for the minimum sequence identity and alignment coverage calculated over either the query or subject sequence. The user may also specify the database against which the search is performed and the type of BLAST program used. The web-server output of BLANNOTATOR has a hierarchical structure (see Figure 6). For each query sequence, the DE with the highest score is shown first. Clicking the link opens a viewer in which the highest scoring DE of each hit set is shown. Hit sets are shown in descending priority order. This second level of the hierarchy also displays alternative protein function predictions, as different clusters consist of sequence matches that do not share common annotations. By opening the third level of hierarchy, the content of each group can be examined in depth. The view reflects the state of the annotation process before GO-based clustering has been applied. This level also shows all different DEs belonging to a certain group. At this level, the user may also see the frequency of each DE, the sum of bit scores of database hits associated with the DE and the UniProt identifier of the most significant match containing the DE.

### Modified Levenshtein distance (mLD) metric

The level of similarity of two DEs was measured using a Levenshtein distance (LD)-based metric. Typically, LD is calculated using a dynamic programming algorithm matching two strings over their full length and giving the minimum number of character differences between the two strings [27]. This means that two annotations with the same set of words but that are in a different order cannot receive the best score. Here, we have modified the computation to deal with changes in word order. Annotations (X and Y) are deconstructed into word (x *_1 _*... x *_n _*and y *_1 _*... y *_n_*) and consecutive word-pair (x *_1,2 _*... x *_n-1,n _*and y *_1,2 _*... y *_n-1,n_*) elements. LD is then computed between the elements of × and Y, and the pair (xy_min_) yielding the smallest LD is recorded. The algorithm then removes xy_min _and all other elements of × and Y that share the words in xy_min _and finds a new xy_min_. The process is repeated until all of the elements of either × or Y have been used. Next, the possible remaining elements of × or Y are scored against an empty string. The mLD between × and Y is then defined as the sum of the LDs of all recorded xy_min_, divided by the length of × or Y, whichever is longer. Here, the normal LD was calculated with a dynamic programming algorithm in which gaps, substitutions and matches are scored as -1, -1 and +2, respectively.

The mLD ranges from zero (similar) to one (dissimilar) and indicates the fraction of character differences between words of two annotations. The metric is independent of word order and considers annotations like '*DNA gyrase, subunit A*' and '*DNA gyrase, A subunit*' to be similar because the only difference between them is the order of their words. On the other hand, if a single word differs between annotations, the mLD is approximately 0.25, *e.g*., in '*Nitric oxide synthase oxygenase*' and '*Neuronal nitric oxide synthase*'. A value of 0.50 indicates that half of the characters between the annotations differ. This is the level of mLD that was typically seen between the annotations of a query and its BLAST hits in the SWISS-PROT data; the average mLDs for query-subject annotation-pairs listed in the evaluation data before and after the regression operation were 0.61 and 0.48, respectively. An mLD of approximately 0.75 represents a limited textual resemblance, like that of '*Cryptochrome DASH*' and ' *DNA photolyase*'. Because different releases of UniProt reported the enzyme commission codes differently, enzyme codes were trimmed from annotations before their similarity was calculated. If one of the DEs was an empty string (because enzyme codes had been removed) or contained only uninformative words (the same set of words as in BLANNOTATOR), the mLD was set to one.

### SWISS-PROT evaluation data, query sequences

Figure 1 outlines the process used to build the evaluation data. Entries in the SWISS-PROT database [3] were downloaded in October 2010 and were considered to contain accurate protein function data. The construction of the evaluation data began by removing entries that were not of bacterial origin or were added to SWISS-PROT prior to 2005. Other derivatives of the primary entry were also tracked, and the primary entry was discarded if any of its derivate entries (given in the accession number-field of the primary entry) did not fulfil our search date criteria. For the remaining set of entries, all older entry versions were extracted, and the first appearance of each DE in each database was recorded. Again, information of alternative entries was taken into account. Older entry versions were retrieved with the help of UniSave [25]. All primary entries containing a DE with the words '*uncharacterized*' or '*UPF*' (*i.e.*, uncharacterised protein family) were then discarded. Next, we selected all active entries of which the first versions were added directly to the SWISS-PROT database or that had emerged from a TrEMBL entry with a different annotation. The mLD method and a distance threshold of 0.50 were used to judge whether a TrEMBL annotation differed from that given in the active SWISS-PROT entry and had underwent re-annotation. If an older entry was found to carry overly similar annotation, this and all more recent entries were flagged. This filtering step was performed mainly to remove candidate test proteins that most likely had been annotated using information originating from SWISS-PROT and to avoid the selection of test sequences for which the function was from the same source as that of its database matches. This had only a marginal effect on the size of the final dataset (1047 DEs were rejected). Next, duplicate annotations between entries were searched, a random entry was chosen for each different DE (from which the name of the gene, *e.g*., '*yclE*', '*ydjP*', '*yfhM*' or '*yisY*', had been removed) and the annotation date (*i.e*., the date at which the entry was added to the SWISS-PROT or the date at which the re-annotation was performed) was recorded. Finally, test sequences with significant sequence similarity to other test sequences (sequence identity ≥ 90% and alignment coverage over the length of the query or subject sequence ≥ 90%) were clustered together, and a random entry was selected from the cluster. The rationale behind this data processing step was to discard proteins that were likely to be involved in the same function but were annotated differently because of synonymous wording or misspelling. For example, duplicated genes can have running numbers in their DE that produce two different annotations.

### SWISS-PROT evaluation data, subject sequences

Test sequences were compared against UniProt using BLAST with default settings [3,16]. BLAST alignments with bit scores smaller than 50 and lengths smaller than 30 were removed, and only the most significant occurrence of each query-subject pair was kept. Matches to the protein sequence itself were also discarded. The number of circularly referenced protein annotations was then minimised by restoring the data to the closest version prior to the characterisation of the query protein. At first, BLAST hits to sequences with a creation data were newer than or identical with the recorded annotation date of the query sequence were removed. The DEs of the remaining matches were reverted to their states preceding the query sequence annotation date with UniSave [25]. In addition to this entire dataset, another version of the dataset was created by removing all BLAST hits with ≥50% sequence identity to the query sequence.

### Calculation of general statistics

The similarities of DEs in the SWISS-PROT data were quantified in terms of the mLD. A score of 0.00 defined annotation pairs with analogous functional descriptions. GO annotations were also tested. Two GO annotations were considered to match when they contained the same set of GO terms. GO term comparisons were done twice. At first using all GO terms and then using only GO terms with experimental (EXP, IDA, IPI, IMP, IGI and IEP) or computational analysis evidence codes (ISS, ISO, ISA, ISM, IGC, IBA, IBD, IKR, IRD and RCA). The latter set of evidence codes comprises human reviewed predictions. For each test protein, we computed the fraction of BLAST hits with a matching GO or DE annotation. Likewise, fractions were counted from BLAST hits with a matching GO and a matching DE annotation (DE and GO dataset) and from sequences with one matching annotation irrespective to the type of the annotation (DE or GO dataset). The size of the BLANNOTATOR sequence group of each correctly annotated BLAST hit (either GO or DE annotation) was recorded, and the maximum of the values was taken. BLANNOTATOR sequence groups were extracted from runs in which all sequence hits were accepted and undesired GO terms had been removed. The mean of the fractions of all 3090 test sequences was calculated.

The similarity between the query sequence annotations and their subject sequence annotations was recorded in terms of mLD. For each query sequence, the minimum, maximum and median quality values were recorded, and the mean and standard deviation of the mLDs were calculated over test sequences having at least one BLAST hit. These statistics were recalculated after discarding BLAST hits below a given sequence identity and alignment coverage (calculated over the subject sequence) threshold combination. Six thresholds (0%, 20%, 40%, 60%, 80% and 100%) were tested, resulting in the testing of 36 (six times six) combinations.

### Competing methods used to analyse the SWISS-PROT data

In addition to BLANNOTATOR, the SWISS-PROT data were analysed with five other methods. The function was inferred from the highest scoring (bit score) non-identical BLAST hit as well as from the top non-identical BLAST match with an annotation that did not contain any uninformative words. The same set of uninformative words that was used for BLANNOTATOR was used here. The three other methods we used were built upon selecting the most common DE in the BLAST hit list, selecting the DE associated with sequences producing the highest sum of bit scores and selecting the DE with the highest word-based score [8]. In addition, the five competing methods were applied to the data so that the information from the largest group of database hits sharing the same GO terms was only given to them. If the largest group of sequence hits sharing the same GO terms contained less than five BLAST hits, the DE was predicted using all matches. Ties were broken randomly.

### Performance evaluation

The similarity between the predicted protein function and the correct annotation in the SWISS-PROT data was measured with the mLD metric, and the means and standard deviations of the mLDs were calculated over the test sequences with at least one BLAST hit. Furthermore, the obtained mLDs were standardised according to the expected mean and standard deviation of the prediction. For a test sequence, given the mLD of its prediction (*x*) and the mean (μ) and standard deviation (σ) of the mLDs of the DE associated with its BLAST hits, the Z-score was calculated as (*x *-μ)/σ. The obtained Z-score was multiplied by negative one so that positive Z-scores indicate improvement and negative scores indicate a decrease in quality. The mean and standard deviation of the Z-scores were calculated from test sequences having at least one BLAST hit. Similarly to computing the general statistics, both statistics described above were recalculated after discarding BLAST hits at a given sequence identity and alignment coverage (calculated over the subject sequence) threshold combination. The same 36 combinations were tested.

### Genome analysis

The protein-coding sequences of the *L. crispatus *strain ST1 [EMBL: FN692037] [26] were characterised with BLANNOTATOR, RAST [20] and inference from the top BLAST match. The sequence similarity search was performed with BLAST against UniProt with default settings, except that alignments were reported for 500 top matches [16,3]. Dubious hits were removed by discarding BLAST hits with bit scores smaller than 50, alignment lengths smaller than 30 and matches with a less than 40% sequence identity or less than 50% alignment coverage (calculated over the subject sequence) to the query sequence. In cases where a query sequence matched the same subject sequence multiple times, only the best scoring (bit score) query-subject pair was retained. BLANNOTATOR was applied to the BLAST data with default parameters, as described in the first section of the Materials and Methods. RAST was also run with default parameters [20]. For the BLAST-based approach, sequences identified by BLAST were sorted by bit score. Protein function predictions were manually compared against each other and against the original annotations that had been created using a wider repertoire of bioinformatics approaches and had been examined carefully during the original study [26].

## List of abbrevations

GO: Gene Ontology; DE: single-line description of protein function; COG: cluster of orthologous sequences; HAMAP: High-quality Automated and Manual Annotation of microbial Proteomes; BLAST: Basic Local Alignment Search Tool; KEGG: Kyoto Encyclopedia of Genes and Genomes; KAAS: KEGG Automatic Annotation Server; RAST: Rapid Annotation using Subsystems Technology; GOA: Gene Ontology annotation database; LD: Levenshtein distance, mLD: modified Levenshtein distance; CRISPR: clustered regularly interspaced short palindromic repeats.

## Competing interests

The authors declare that they have no competing interests.

## Authors' contributions

MK invented, designed and wrote the algorithm, collected the data, performed the data analysis and drafted the manuscript. TO participated in the data analysis, performed the manual analysis of the *L. crispatus *strain ST1 proteome annotations and participated in the drafting of the manuscript. LH supervised and financed the study and participated in the drafting of the manuscript. All authors approved the final manuscript. We thank the Bioinformatics group for helpful discussions of this project.

## Supplementary Material

Additional file 1 **This file contains the supporting tables and figures regarding the analysis of the SWISS-PROT data**. Click here for file
