# Supplementary material for: BLANNOTATOR: enhanced homology-based function prediction of bacterial proteins
Source: BMC Bioinformatics. 2012 Feb 15;13:33. doi: 10.1186/1471-2105-13-33 (PMC3386020; doi:10.1186/1471-2105-13-33)
Supplement: Additional file 1 — This file contains the supporting tables and figures regarding the analysis of the SWISS-PROT data. [file 1471-2105-13-33-S1.DOC]

# Supplementary Material for “BLANNOTATOR: enhanced homology-based function prediction of bacterial proteins”

Matti Kankainen^1,2^*, Teija Ojala^1^, and Liisa Holm^1,3^

**
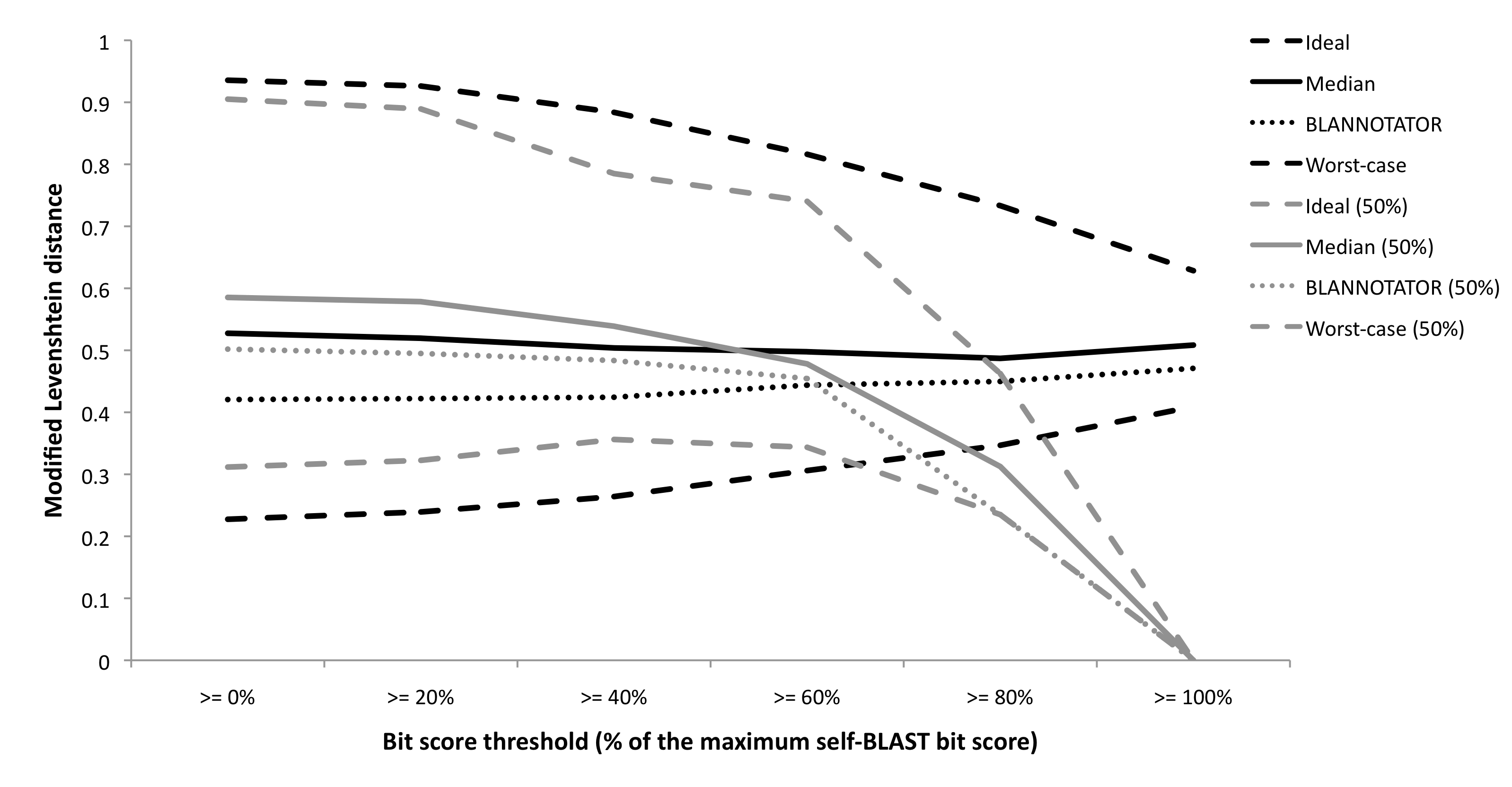
**

**Figure S1.** Mean annotation quality of the ideal and worst-case predictions in Swiss-Prot dataset. Modified Levenshtein distance-based statistics is shown for the ideal (dash), median (solid) and worst-case (dash) predictions after removing BLAST hits at various relative bit score thresholds. Data is also shown for BLANNOTATOR predictions (dotted line). Relative bit score is the obtained bit score divided by query’s self-BLAST bit score. Modified Levenshtein distance-based statistics was used to measure the annotation quality. Grey lines show results after removal of BLAST hits with sequence identity greater than 50% to the query sequence.

**
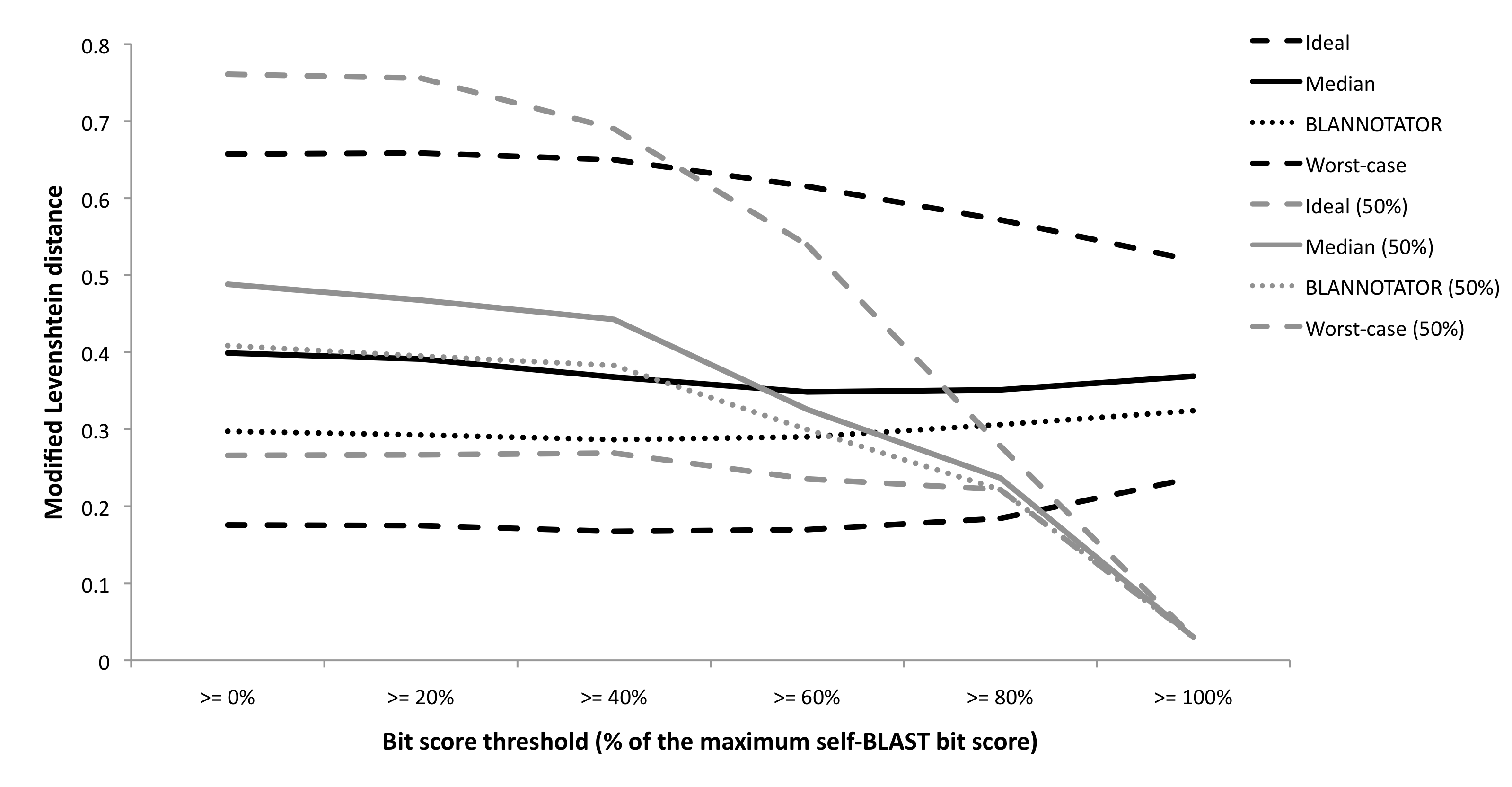
**

**Figure S2.** Notations as in Figure S1. Function prediction was done from data having circularly referenced annotations.


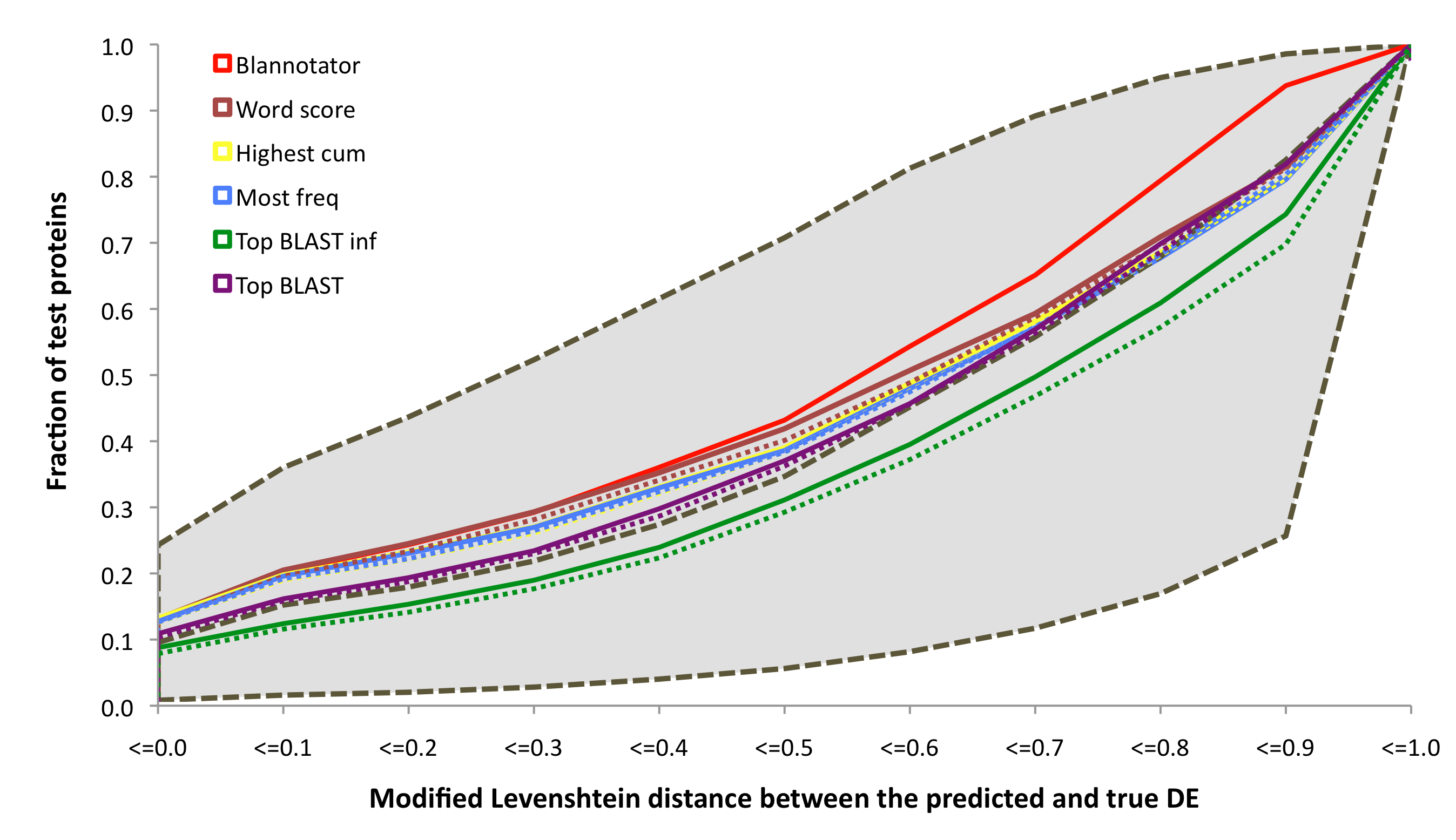
**Figure S3.** The quality of automated protein function predictions. The fraction of predictions below a certain modified Levenshtein distance from the correct annotation is shown. Function prediction was based on the most significant BLAST match (purple), the top BLAST match without any uninformative words (green), the most common annotation among BLAST hits (blues), the annotation associated with the highest bit score sum (yellow), a word-based scoring scheme (brown) and BLANNOTATOR (red). Dashed lines show the performance of the tool when applied to the largest group of matches sharing a common GO annotation. The black dashed lines and the grey background indicates the theoretical level of performance when the ideal, median or worst-case predictions were chosen. Function prediction was done after removal of BLAST hits with sequence identity greater than 50% to the query sequence.


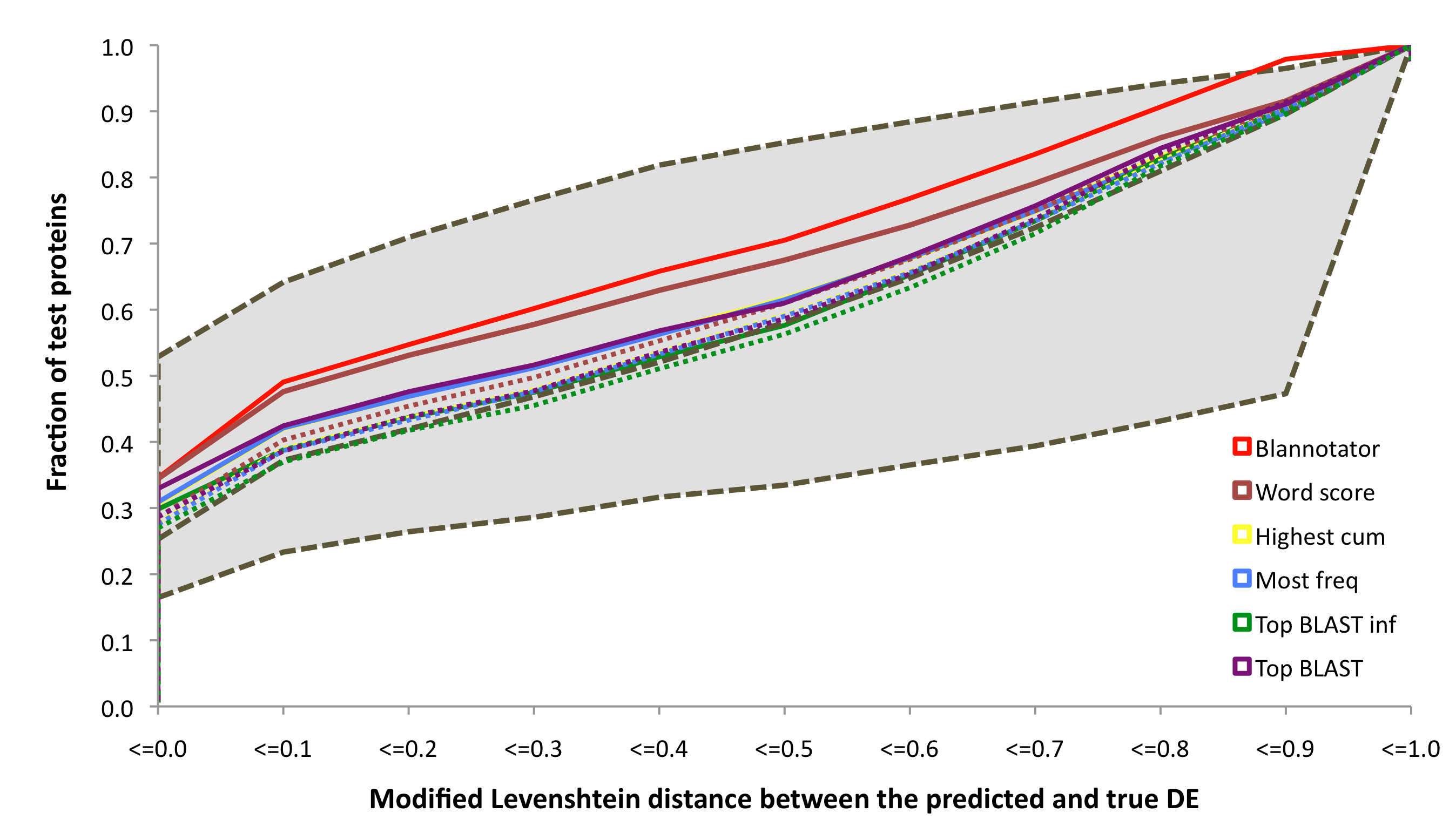
**Figure S4.** Notations as in Figure S3. Function prediction was done from data having circularly referenced annotations.

**
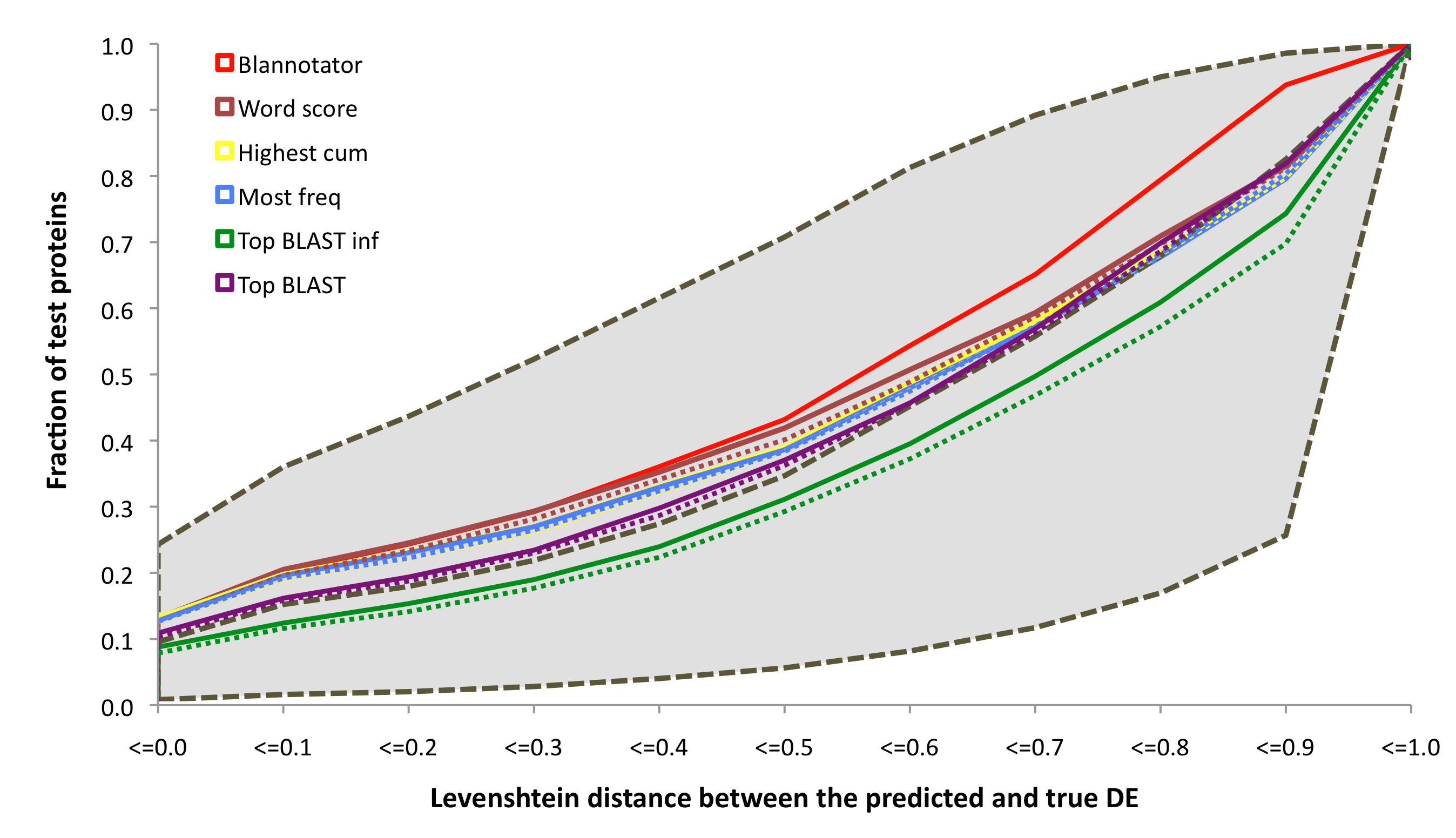
**

**Figure S5.** Notations as in Figure S3. Function prediction was done from data having circularly referenced annotations, but after removal of BLAST hits with sequence identity greater than 50% to the query sequence.

|  |  |  | Ideal | |  | Median | |  | Worst | |
| --- | --- | --- | --- | --- | --- | --- | --- | --- | --- | --- |
| ID% | COV% | Hits | mean | sd |  | mean | sd |  | mean | sd |
| 0 | 0 | 3007 | 0.23 | 0.26 |  | 0.53 | 0.34 |  | 0.94 | 0.16 |
| 0 | 20 | 3002 | 0.23 | 0.26 |  | 0.53 | 0.34 |  | 0.94 | 0.16 |
| 0 | 40 | 2994 | 0.23 | 0.26 |  | 0.52 | 0.34 |  | 0.93 | 0.16 |
| 0 | 60 | 2986 | 0.23 | 0.27 |  | 0.52 | 0.35 |  | 0.93 | 0.16 |
| 0 | 80 | 2952 | 0.24 | 0.27 |  | 0.52 | 0.35 |  | 0.92 | 0.17 |
| 0 | 100 | 1766 | 0.33 | 0.32 |  | 0.50 | 0.34 |  | 0.77 | 0.27 |
| 20 | 0 | 3007 | 0.23 | 0.26 |  | 0.53 | 0.34 |  | 0.94 | 0.16 |
| 20 | 20 | 3002 | 0.23 | 0.26 |  | 0.53 | 0.34 |  | 0.94 | 0.16 |
| 20 | 40 | 2994 | 0.23 | 0.26 |  | 0.52 | 0.34 |  | 0.93 | 0.16 |
| 20 | 60 | 2986 | 0.23 | 0.27 |  | 0.52 | 0.35 |  | 0.93 | 0.16 |
| 20 | 80 | 2952 | 0.24 | 0.27 |  | 0.52 | 0.35 |  | 0.92 | 0.17 |
| 20 | 100 | 1766 | 0.33 | 0.32 |  | 0.50 | 0.34 |  | 0.77 | 0.27 |
| 40 | 0 | 2959 | 0.25 | 0.29 |  | 0.51 | 0.35 |  | 0.90 | 0.19 |
| 40 | 20 | 2952 | 0.25 | 0.29 |  | 0.51 | 0.35 |  | 0.90 | 0.19 |
| 40 | 40 | 2944 | 0.26 | 0.29 |  | 0.50 | 0.35 |  | 0.90 | 0.19 |
| 40 | 60 | 2930 | 0.26 | 0.29 |  | 0.50 | 0.35 |  | 0.90 | 0.19 |
| 40 | 80 | 2889 | 0.26 | 0.29 |  | 0.50 | 0.35 |  | 0.89 | 0.20 |
| 40 | 100 | 1737 | 0.33 | 0.32 |  | 0.50 | 0.34 |  | 0.76 | 0.28 |
| 60 | 0 | 2623 | 0.31 | 0.32 |  | 0.50 | 0.36 |  | 0.82 | 0.25 |
| 60 | 20 | 2619 | 0.31 | 0.32 |  | 0.50 | 0.36 |  | 0.82 | 0.25 |
| 60 | 40 | 2611 | 0.31 | 0.32 |  | 0.50 | 0.36 |  | 0.82 | 0.26 |
| 60 | 60 | 2597 | 0.31 | 0.33 |  | 0.50 | 0.36 |  | 0.82 | 0.26 |
| 60 | 80 | 2563 | 0.32 | 0.33 |  | 0.50 | 0.36 |  | 0.81 | 0.26 |
| 60 | 100 | 1579 | 0.35 | 0.34 |  | 0.51 | 0.35 |  | 0.75 | 0.29 |
| 80 | 0 | 2005 | 0.35 | 0.34 |  | 0.49 | 0.36 |  | 0.72 | 0.31 |
| 80 | 20 | 2004 | 0.35 | 0.34 |  | 0.49 | 0.36 |  | 0.72 | 0.31 |
| 80 | 40 | 1995 | 0.35 | 0.34 |  | 0.49 | 0.36 |  | 0.72 | 0.31 |
| 80 | 60 | 1981 | 0.35 | 0.34 |  | 0.49 | 0.36 |  | 0.72 | 0.31 |
| 80 | 80 | 1957 | 0.35 | 0.34 |  | 0.49 | 0.36 |  | 0.71 | 0.32 |
| 80 | 100 | 1297 | 0.36 | 0.34 |  | 0.50 | 0.35 |  | 0.70 | 0.31 |
| 100 | 0 | 395 | 0.40 | 0.35 |  | 0.50 | 0.36 |  | 0.62 | 0.35 |
| 100 | 20 | 394 | 0.40 | 0.35 |  | 0.50 | 0.36 |  | 0.62 | 0.35 |
| 100 | 40 | 393 | 0.40 | 0.35 |  | 0.50 | 0.36 |  | 0.62 | 0.35 |
| 100 | 60 | 390 | 0.40 | 0.35 |  | 0.50 | 0.36 |  | 0.62 | 0.35 |
| 100 | 80 | 385 | 0.40 | 0.35 |  | 0.50 | 0.36 |  | 0.62 | 0.35 |
| 100 | 100 | 198 | 0.44 | 0.35 |  | 0.52 | 0.35 |  | 0.63 | 0.34 |

**Table S1.** Effects of sequence filtering. Shown are the mean and standard deviation of the minimum (the best achievable result for the homology-based transfer), median, maximum (the worst-case result for the homology-based transfer) mLDs of each test sequence. Statistics were calculated several times after removal of BLAST hits with smaller than the given sequence identity (ID%) or alignment coverage (COV%) to the query sequence and reported values was calculated over set of test sequences having at least one BLAST hit (Hits)

|  |  |  | Ideal | |  | Median | |  | Worst | |
| --- | --- | --- | --- | --- | --- | --- | --- | --- | --- | --- |
| ID% | COV% | Hits | mean | sd |  | mean | sd |  | mean | sd |
| 0 | 0 | 3021 | 0.18 | 0.28 |  | 0.40 | 0.36 |  | 0.66 | 0.42 |
| 0 | 20 | 3020 | 0.18 | 0.28 |  | 0.40 | 0.36 |  | 0.66 | 0.42 |
| 0 | 40 | 3017 | 0.18 | 0.28 |  | 0.40 | 0.36 |  | 0.66 | 0.42 |
| 0 | 60 | 3013 | 0.18 | 0.28 |  | 0.40 | 0.36 |  | 0.65 | 0.42 |
| 0 | 80 | 3001 | 0.17 | 0.27 |  | 0.39 | 0.36 |  | 0.65 | 0.42 |
| 0 | 100 | 2057 | 0.18 | 0.27 |  | 0.36 | 0.36 |  | 0.62 | 0.38 |
| 20 | 0 | 3021 | 0.18 | 0.28 |  | 0.40 | 0.36 |  | 0.66 | 0.42 |
| 20 | 20 | 3020 | 0.18 | 0.28 |  | 0.40 | 0.36 |  | 0.66 | 0.42 |
| 20 | 40 | 3017 | 0.18 | 0.28 |  | 0.40 | 0.36 |  | 0.66 | 0.42 |
| 20 | 60 | 3013 | 0.18 | 0.28 |  | 0.40 | 0.36 |  | 0.65 | 0.42 |
| 20 | 80 | 3001 | 0.17 | 0.27 |  | 0.39 | 0.36 |  | 0.65 | 0.42 |
| 20 | 100 | 2057 | 0.18 | 0.27 |  | 0.36 | 0.36 |  | 0.62 | 0.38 |
| 40 | 0 | 3011 | 0.17 | 0.27 |  | 0.38 | 0.36 |  | 0.66 | 0.41 |
| 40 | 20 | 3009 | 0.17 | 0.27 |  | 0.38 | 0.36 |  | 0.66 | 0.41 |
| 40 | 40 | 3006 | 0.17 | 0.27 |  | 0.38 | 0.36 |  | 0.65 | 0.41 |
| 40 | 60 | 2999 | 0.17 | 0.27 |  | 0.38 | 0.36 |  | 0.65 | 0.41 |
| 40 | 80 | 2983 | 0.17 | 0.27 |  | 0.37 | 0.36 |  | 0.65 | 0.41 |
| 40 | 100 | 2026 | 0.18 | 0.27 |  | 0.36 | 0.35 |  | 0.61 | 0.39 |
| 60 | 0 | 2868 | 0.17 | 0.26 |  | 0.37 | 0.36 |  | 0.63 | 0.40 |
| 60 | 20 | 2865 | 0.17 | 0.26 |  | 0.37 | 0.36 |  | 0.63 | 0.40 |
| 60 | 40 | 2862 | 0.17 | 0.27 |  | 0.37 | 0.36 |  | 0.63 | 0.40 |
| 60 | 60 | 2854 | 0.17 | 0.27 |  | 0.36 | 0.36 |  | 0.62 | 0.40 |
| 60 | 80 | 2833 | 0.17 | 0.27 |  | 0.36 | 0.36 |  | 0.62 | 0.40 |
| 60 | 100 | 1908 | 0.19 | 0.27 |  | 0.36 | 0.35 |  | 0.59 | 0.39 |
| 80 | 0 | 2526 | 0.19 | 0.28 |  | 0.35 | 0.35 |  | 0.58 | 0.39 |
| 80 | 20 | 2526 | 0.19 | 0.28 |  | 0.35 | 0.35 |  | 0.58 | 0.39 |
| 80 | 40 | 2518 | 0.19 | 0.28 |  | 0.35 | 0.35 |  | 0.57 | 0.39 |
| 80 | 60 | 2510 | 0.19 | 0.28 |  | 0.35 | 0.35 |  | 0.57 | 0.39 |
| 80 | 80 | 2490 | 0.19 | 0.28 |  | 0.35 | 0.35 |  | 0.57 | 0.39 |
| 80 | 100 | 1717 | 0.20 | 0.28 |  | 0.35 | 0.35 |  | 0.55 | 0.39 |
| 100 | 0 | 664 | 0.24 | 0.32 |  | 0.35 | 0.36 |  | 0.49 | 0.39 |
| 100 | 20 | 663 | 0.24 | 0.32 |  | 0.35 | 0.36 |  | 0.49 | 0.39 |
| 100 | 40 | 660 | 0.24 | 0.32 |  | 0.35 | 0.36 |  | 0.49 | 0.39 |
| 100 | 60 | 657 | 0.24 | 0.32 |  | 0.35 | 0.35 |  | 0.49 | 0.39 |
| 100 | 80 | 650 | 0.24 | 0.32 |  | 0.35 | 0.35 |  | 0.48 | 0.39 |
| 100 | 100 | 330 | 0.22 | 0.31 |  | 0.31 | 0.35 |  | 0.45 | 0.39 |

**Table S2.** Notations as in Table S1. Function prediction was done from data having circularly referenced annotations.

|  |  |  | Ideal | |  | Median | |  | Worst | |
| --- | --- | --- | --- | --- | --- | --- | --- | --- | --- | --- |
| ID% | COV% | Hits | mean | sd |  | mean | sd |  | mean | sd |
| 0 | 0 | 2313 | 0.31 | 0.28 |  | 0.59 | 0.32 |  | 0.91 | 0.20 |
| 0 | 20 | 2307 | 0.31 | 0.28 |  | 0.58 | 0.32 |  | 0.90 | 0.20 |
| 0 | 40 | 2292 | 0.31 | 0.29 |  | 0.58 | 0.33 |  | 0.90 | 0.20 |
| 0 | 60 | 2271 | 0.32 | 0.29 |  | 0.58 | 0.33 |  | 0.90 | 0.20 |
| 0 | 80 | 2210 | 0.33 | 0.30 |  | 0.58 | 0.33 |  | 0.88 | 0.21 |
| 0 | 100 | 524 | 0.43 | 0.32 |  | 0.54 | 0.33 |  | 0.67 | 0.31 |
| 20 | 0 | 2313 | 0.31 | 0.28 |  | 0.59 | 0.32 |  | 0.91 | 0.20 |
| 20 | 20 | 2307 | 0.31 | 0.28 |  | 0.58 | 0.32 |  | 0.90 | 0.20 |
| 20 | 40 | 2292 | 0.31 | 0.29 |  | 0.58 | 0.33 |  | 0.90 | 0.20 |
| 20 | 60 | 2271 | 0.32 | 0.29 |  | 0.58 | 0.33 |  | 0.90 | 0.20 |
| 20 | 80 | 2210 | 0.33 | 0.30 |  | 0.58 | 0.33 |  | 0.88 | 0.21 |
| 20 | 100 | 524 | 0.43 | 0.32 |  | 0.54 | 0.33 |  | 0.67 | 0.31 |
| 40 | 0 | 2031 | 0.35 | 0.31 |  | 0.56 | 0.34 |  | 0.84 | 0.24 |
| 40 | 20 | 2022 | 0.35 | 0.31 |  | 0.56 | 0.34 |  | 0.84 | 0.24 |
| 40 | 40 | 1998 | 0.35 | 0.31 |  | 0.55 | 0.34 |  | 0.83 | 0.24 |
| 40 | 60 | 1955 | 0.35 | 0.31 |  | 0.55 | 0.34 |  | 0.83 | 0.25 |
| 40 | 80 | 1871 | 0.35 | 0.31 |  | 0.55 | 0.34 |  | 0.82 | 0.25 |
| 40 | 100 | 403 | 0.42 | 0.32 |  | 0.51 | 0.33 |  | 0.64 | 0.32 |

**Table 3.** Notations as in Table S1. Function prediction was done after removal of BLAST hits with sequence identity greater than 50% to the query sequence.

|  |  |  | Ideal | |  | Median | |  | Worst | |
| --- | --- | --- | --- | --- | --- | --- | --- | --- | --- | --- |
| ID% | COV% | Hits | mean | sd |  | mean | sd |  | mean | sd |
| 0 | 0 | 2395 | 0.27 | 0.31 |  | 0.49 | 0.36 |  | 0.76 | 0.36 |
| 0 | 20 | 2391 | 0.27 | 0.31 |  | 0.49 | 0.36 |  | 0.76 | 0.36 |
| 0 | 40 | 2382 | 0.27 | 0.31 |  | 0.48 | 0.35 |  | 0.76 | 0.36 |
| 0 | 60 | 2365 | 0.27 | 0.31 |  | 0.48 | 0.36 |  | 0.76 | 0.36 |
| 0 | 80 | 2333 | 0.26 | 0.31 |  | 0.47 | 0.36 |  | 0.75 | 0.37 |
| 0 | 100 | 842 | 0.35 | 0.33 |  | 0.48 | 0.35 |  | 0.61 | 0.36 |
| 20 | 0 | 2395 | 0.27 | 0.31 |  | 0.49 | 0.36 |  | 0.76 | 0.36 |
| 20 | 20 | 2391 | 0.27 | 0.31 |  | 0.49 | 0.36 |  | 0.76 | 0.36 |
| 20 | 40 | 2382 | 0.27 | 0.31 |  | 0.48 | 0.36 |  | 0.76 | 0.36 |
| 20 | 60 | 2365 | 0.27 | 0.31 |  | 0.48 | 0.36 |  | 0.76 | 0.36 |
| 20 | 80 | 2333 | 0.26 | 0.31 |  | 0.47 | 0.36 |  | 0.75 | 0.37 |
| 20 | 100 | 842 | 0.35 | 0.33 |  | 0.48 | 0.35 |  | 0.61 | 0.36 |
| 40 | 0 | 2251 | 0.27 | 0.30 |  | 0.47 | 0.36 |  | 0.74 | 0.36 |
| 40 | 20 | 2244 | 0.27 | 0.30 |  | 0.46 | 0.35 |  | 0.73 | 0.36 |
| 40 | 40 | 2228 | 0.27 | 0.30 |  | 0.46 | 0.36 |  | 0.73 | 0.36 |
| 40 | 60 | 2200 | 0.27 | 0.30 |  | 0.46 | 0.35 |  | 0.73 | 0.36 |
| 40 | 80 | 2138 | 0.27 | 0.31 |  | 0.45 | 0.36 |  | 0.72 | 0.36 |
| 40 | 100 | 649 | 0.32 | 0.32 |  | 0.44 | 0.35 |  | 0.57 | 0.36 |

**Table S4.** Notations as in Table S1. Function prediction was done from data having circularly referenced annotations, but after removal of BLAST hits with sequence identity greater than 50% to the query sequence.

| ID% | COV% | Hits | Top BLAST | | | | |  | Top BLAST inf | | | | |  | Highest cum | | | | |  | Most freq | | | | |  | Word-score | | | | |  | BLANNOTATOR | |
| --- | --- | --- | --- | --- | --- | --- | --- | --- | --- | --- | --- | --- | --- | --- | --- | --- | --- | --- | --- | --- | --- | --- | --- | --- | --- | --- | --- | --- | --- | --- | --- | --- | --- | --- |
|  |  |  | ALL | |  | GO | |  | ALL | |  | GO | |  | ALL | |  | GO | |  | ALL | |  | GO | |  | ALL | |  | GO | |  |  |  |
|  |  |  | mean | sd |  | mean | sd |  | mean | sd |  | mean | sd |  | mean | sd |  | mean | sd |  | mean | sd |  | mean | sd |  | mean | sd |  | mean | sd |  | mean | sd |
| 0 | 0 | 3007 | 0.54 | 0.36 |  | 0.54 | 0.35 |  | 0.60 | 0.35 |  | 0.62 | 0.35 |  | 0.47 | 0.37 |  | 0.49 | 0.36 |  | 0.48 | 0.37 |  | 0.49 | 0.36 |  | 0.46 | 0.37 |  | 0.48 | 0.36 |  | 0.42 | 0.34 |
| 0 | 20 | 3002 | 0.54 | 0.35 |  | 0.54 | 0.35 |  | 0.60 | 0.35 |  | 0.62 | 0.35 |  | 0.47 | 0.37 |  | 0.49 | 0.36 |  | 0.48 | 0.37 |  | 0.49 | 0.36 |  | 0.45 | 0.37 |  | 0.48 | 0.36 |  | 0.42 | 0.34 |
| 0 | 40 | 2994 | 0.54 | 0.36 |  | 0.53 | 0.35 |  | 0.60 | 0.35 |  | 0.62 | 0.35 |  | 0.47 | 0.37 |  | 0.49 | 0.36 |  | 0.47 | 0.37 |  | 0.49 | 0.36 |  | 0.45 | 0.37 |  | 0.48 | 0.36 |  | 0.42 | 0.34 |
| 0 | 60 | 2986 | 0.54 | 0.35 |  | 0.54 | 0.35 |  | 0.60 | 0.36 |  | 0.62 | 0.35 |  | 0.46 | 0.37 |  | 0.49 | 0.36 |  | 0.47 | 0.37 |  | 0.48 | 0.36 |  | 0.45 | 0.37 |  | 0.48 | 0.37 |  | 0.42 | 0.34 |
| 0 | 80 | 2952 | 0.53 | 0.36 |  | 0.54 | 0.35 |  | 0.60 | 0.36 |  | 0.62 | 0.36 |  | 0.46 | 0.37 |  | 0.49 | 0.36 |  | 0.47 | 0.37 |  | 0.49 | 0.36 |  | 0.45 | 0.37 |  | 0.48 | 0.36 |  | 0.42 | 0.34 |
| 0 | 100 | 1766 | 0.52 | 0.35 |  | 0.52 | 0.35 |  | 0.61 | 0.38 |  | 0.62 | 0.38 |  | 0.48 | 0.36 |  | 0.49 | 0.35 |  | 0.47 | 0.35 |  | 0.48 | 0.35 |  | 0.47 | 0.36 |  | 0.48 | 0.36 |  | 0.46 | 0.34 |
| 20 | 0 | 3007 | 0.54 | 0.36 |  | 0.54 | 0.35 |  | 0.60 | 0.35 |  | 0.62 | 0.35 |  | 0.47 | 0.37 |  | 0.49 | 0.36 |  | 0.48 | 0.37 |  | 0.49 | 0.36 |  | 0.46 | 0.37 |  | 0.48 | 0.37 |  | 0.42 | 0.34 |
| 20 | 20 | 3002 | 0.54 | 0.35 |  | 0.54 | 0.35 |  | 0.60 | 0.35 |  | 0.61 | 0.35 |  | 0.47 | 0.37 |  | 0.49 | 0.36 |  | 0.48 | 0.37 |  | 0.49 | 0.36 |  | 0.45 | 0.37 |  | 0.48 | 0.36 |  | 0.42 | 0.34 |
| 20 | 40 | 2994 | 0.54 | 0.36 |  | 0.53 | 0.35 |  | 0.60 | 0.35 |  | 0.61 | 0.35 |  | 0.47 | 0.37 |  | 0.49 | 0.36 |  | 0.47 | 0.37 |  | 0.48 | 0.36 |  | 0.45 | 0.37 |  | 0.48 | 0.36 |  | 0.42 | 0.34 |
| 20 | 60 | 2986 | 0.54 | 0.35 |  | 0.53 | 0.35 |  | 0.60 | 0.36 |  | 0.62 | 0.35 |  | 0.46 | 0.37 |  | 0.48 | 0.36 |  | 0.47 | 0.37 |  | 0.48 | 0.36 |  | 0.45 | 0.37 |  | 0.48 | 0.37 |  | 0.42 | 0.34 |
| 20 | 80 | 2952 | 0.53 | 0.36 |  | 0.54 | 0.35 |  | 0.60 | 0.36 |  | 0.62 | 0.35 |  | 0.46 | 0.37 |  | 0.48 | 0.36 |  | 0.47 | 0.37 |  | 0.48 | 0.36 |  | 0.45 | 0.37 |  | 0.48 | 0.36 |  | 0.42 | 0.34 |
| 20 | 100 | 1766 | 0.52 | 0.35 |  | 0.52 | 0.35 |  | 0.61 | 0.38 |  | 0.62 | 0.38 |  | 0.48 | 0.36 |  | 0.49 | 0.35 |  | 0.47 | 0.35 |  | 0.48 | 0.35 |  | 0.47 | 0.36 |  | 0.48 | 0.36 |  | 0.46 | 0.34 |
| 40 | 0 | 2959 | 0.53 | 0.35 |  | 0.54 | 0.35 |  | 0.61 | 0.36 |  | 0.63 | 0.36 |  | 0.46 | 0.37 |  | 0.48 | 0.36 |  | 0.46 | 0.37 |  | 0.48 | 0.36 |  | 0.45 | 0.37 |  | 0.47 | 0.37 |  | 0.42 | 0.34 |
| 40 | 20 | 2952 | 0.53 | 0.35 |  | 0.53 | 0.35 |  | 0.61 | 0.36 |  | 0.62 | 0.36 |  | 0.46 | 0.37 |  | 0.48 | 0.36 |  | 0.46 | 0.37 |  | 0.48 | 0.36 |  | 0.45 | 0.37 |  | 0.47 | 0.37 |  | 0.42 | 0.34 |
| 40 | 40 | 2944 | 0.53 | 0.35 |  | 0.53 | 0.35 |  | 0.61 | 0.36 |  | 0.63 | 0.36 |  | 0.46 | 0.37 |  | 0.48 | 0.36 |  | 0.46 | 0.37 |  | 0.48 | 0.36 |  | 0.45 | 0.37 |  | 0.47 | 0.37 |  | 0.42 | 0.34 |
| 40 | 60 | 2930 | 0.53 | 0.35 |  | 0.53 | 0.35 |  | 0.61 | 0.36 |  | 0.62 | 0.36 |  | 0.46 | 0.37 |  | 0.48 | 0.36 |  | 0.46 | 0.37 |  | 0.47 | 0.36 |  | 0.45 | 0.37 |  | 0.47 | 0.36 |  | 0.42 | 0.34 |
| 40 | 80 | 2889 | 0.53 | 0.36 |  | 0.53 | 0.35 |  | 0.61 | 0.36 |  | 0.62 | 0.36 |  | 0.46 | 0.37 |  | 0.48 | 0.36 |  | 0.46 | 0.36 |  | 0.47 | 0.36 |  | 0.45 | 0.37 |  | 0.47 | 0.36 |  | 0.42 | 0.34 |
| 40 | 100 | 1737 | 0.51 | 0.35 |  | 0.52 | 0.35 |  | 0.61 | 0.38 |  | 0.62 | 0.38 |  | 0.48 | 0.36 |  | 0.48 | 0.35 |  | 0.47 | 0.35 |  | 0.47 | 0.35 |  | 0.47 | 0.36 |  | 0.48 | 0.35 |  | 0.45 | 0.34 |
| 60 | 0 | 2623 | 0.52 | 0.36 |  | 0.52 | 0.36 |  | 0.63 | 0.37 |  | 0.65 | 0.37 |  | 0.48 | 0.37 |  | 0.48 | 0.37 |  | 0.47 | 0.36 |  | 0.48 | 0.36 |  | 0.47 | 0.37 |  | 0.48 | 0.37 |  | 0.45 | 0.35 |
| 60 | 20 | 2619 | 0.52 | 0.36 |  | 0.52 | 0.35 |  | 0.63 | 0.37 |  | 0.65 | 0.37 |  | 0.48 | 0.37 |  | 0.48 | 0.36 |  | 0.47 | 0.37 |  | 0.48 | 0.36 |  | 0.47 | 0.37 |  | 0.48 | 0.37 |  | 0.45 | 0.35 |
| 60 | 40 | 2611 | 0.52 | 0.36 |  | 0.52 | 0.35 |  | 0.63 | 0.37 |  | 0.65 | 0.37 |  | 0.47 | 0.37 |  | 0.48 | 0.37 |  | 0.47 | 0.36 |  | 0.48 | 0.36 |  | 0.47 | 0.37 |  | 0.48 | 0.37 |  | 0.45 | 0.35 |
| 60 | 60 | 2597 | 0.52 | 0.36 |  | 0.52 | 0.35 |  | 0.63 | 0.37 |  | 0.64 | 0.37 |  | 0.47 | 0.37 |  | 0.48 | 0.37 |  | 0.47 | 0.36 |  | 0.48 | 0.36 |  | 0.47 | 0.37 |  | 0.48 | 0.37 |  | 0.45 | 0.35 |
| 60 | 80 | 2563 | 0.52 | 0.36 |  | 0.52 | 0.35 |  | 0.63 | 0.37 |  | 0.64 | 0.37 |  | 0.47 | 0.37 |  | 0.48 | 0.37 |  | 0.47 | 0.37 |  | 0.48 | 0.36 |  | 0.47 | 0.37 |  | 0.48 | 0.37 |  | 0.45 | 0.35 |
| 60 | 100 | 1579 | 0.51 | 0.35 |  | 0.51 | 0.35 |  | 0.62 | 0.38 |  | 0.63 | 0.39 |  | 0.49 | 0.36 |  | 0.49 | 0.36 |  | 0.48 | 0.36 |  | 0.48 | 0.35 |  | 0.48 | 0.36 |  | 0.48 | 0.36 |  | 0.46 | 0.35 |
| 80 | 0 | 2005 | 0.50 | 0.35 |  | 0.50 | 0.35 |  | 0.64 | 0.38 |  | 0.65 | 0.38 |  | 0.48 | 0.36 |  | 0.48 | 0.37 |  | 0.48 | 0.36 |  | 0.47 | 0.36 |  | 0.47 | 0.37 |  | 0.47 | 0.37 |  | 0.45 | 0.35 |
| 80 | 20 | 2004 | 0.50 | 0.35 |  | 0.50 | 0.35 |  | 0.64 | 0.38 |  | 0.65 | 0.38 |  | 0.48 | 0.36 |  | 0.48 | 0.36 |  | 0.48 | 0.36 |  | 0.48 | 0.36 |  | 0.47 | 0.37 |  | 0.48 | 0.37 |  | 0.45 | 0.35 |
| 80 | 40 | 1995 | 0.49 | 0.35 |  | 0.50 | 0.35 |  | 0.64 | 0.38 |  | 0.65 | 0.38 |  | 0.48 | 0.36 |  | 0.48 | 0.37 |  | 0.47 | 0.36 |  | 0.47 | 0.36 |  | 0.47 | 0.37 |  | 0.47 | 0.37 |  | 0.45 | 0.35 |
| 80 | 60 | 1981 | 0.49 | 0.35 |  | 0.49 | 0.35 |  | 0.64 | 0.38 |  | 0.65 | 0.38 |  | 0.47 | 0.36 |  | 0.47 | 0.36 |  | 0.47 | 0.36 |  | 0.47 | 0.36 |  | 0.47 | 0.37 |  | 0.47 | 0.37 |  | 0.45 | 0.35 |
| 80 | 80 | 1957 | 0.49 | 0.35 |  | 0.49 | 0.35 |  | 0.64 | 0.38 |  | 0.65 | 0.38 |  | 0.47 | 0.36 |  | 0.47 | 0.37 |  | 0.47 | 0.36 |  | 0.47 | 0.36 |  | 0.47 | 0.37 |  | 0.47 | 0.37 |  | 0.45 | 0.35 |
| 80 | 100 | 1297 | 0.50 | 0.35 |  | 0.50 | 0.35 |  | 0.62 | 0.39 |  | 0.62 | 0.39 |  | 0.48 | 0.36 |  | 0.48 | 0.36 |  | 0.48 | 0.36 |  | 0.48 | 0.36 |  | 0.47 | 0.36 |  | 0.47 | 0.36 |  | 0.46 | 0.34 |
| 100 | 0 | 395 | 0.48 | 0.35 |  | 0.48 | 0.35 |  | 0.68 | 0.39 |  | 0.68 | 0.39 |  | 0.48 | 0.37 |  | 0.49 | 0.36 |  | 0.49 | 0.37 |  | 0.49 | 0.36 |  | 0.50 | 0.37 |  | 0.50 | 0.37 |  | 0.46 | 0.35 |
| 100 | 20 | 394 | 0.48 | 0.35 |  | 0.48 | 0.35 |  | 0.68 | 0.39 |  | 0.68 | 0.39 |  | 0.48 | 0.37 |  | 0.48 | 0.37 |  | 0.49 | 0.37 |  | 0.49 | 0.37 |  | 0.50 | 0.37 |  | 0.49 | 0.37 |  | 0.46 | 0.35 |
| 100 | 40 | 393 | 0.48 | 0.35 |  | 0.48 | 0.35 |  | 0.68 | 0.39 |  | 0.68 | 0.39 |  | 0.48 | 0.37 |  | 0.49 | 0.37 |  | 0.48 | 0.37 |  | 0.49 | 0.37 |  | 0.50 | 0.37 |  | 0.50 | 0.37 |  | 0.46 | 0.36 |
| 100 | 60 | 390 | 0.48 | 0.35 |  | 0.48 | 0.35 |  | 0.68 | 0.39 |  | 0.68 | 0.39 |  | 0.48 | 0.37 |  | 0.48 | 0.37 |  | 0.48 | 0.37 |  | 0.48 | 0.37 |  | 0.49 | 0.37 |  | 0.49 | 0.37 |  | 0.46 | 0.35 |
| 100 | 80 | 385 | 0.48 | 0.35 |  | 0.48 | 0.35 |  | 0.67 | 0.39 |  | 0.68 | 0.39 |  | 0.48 | 0.37 |  | 0.49 | 0.37 |  | 0.48 | 0.37 |  | 0.49 | 0.37 |  | 0.50 | 0.37 |  | 0.50 | 0.37 |  | 0.46 | 0.36 |
| 100 | 100 | 198 | 0.52 | 0.34 |  | 0.52 | 0.34 |  | 0.68 | 0.38 |  | 0.69 | 0.38 |  | 0.52 | 0.36 |  | 0.52 | 0.36 |  | 0.52 | 0.36 |  | 0.52 | 0.36 |  | 0.51 | 0.36 |  | 0.52 | 0.36 |  | 0.49 | 0.35 |

**Table S5.** Prediction quality of the tested automated protein function prediction methods. The table shows the mean and standard deviation of mLDs of predictions, calculated over test proteins having at least one BLAST hit. Number of test proteins with at least one BLAST hit is shown in Hits-column. Statistics were calculated several times after removal of BLAST hits with smaller than the given sequence identity (ID%) or alignment coverage (COV%) to the query sequence. DE were predicted based upon the most significant BLAST match (Top BLAST), the most significant BLAST match of which annotation did not had uninformative words (Top BLAST inf), the highest sum of bit scores (Highest cum), the frequency in the hit list (Most freq), the word importance based scoring method (Word-score) and BLANNOTATOR.

|  |  |  | Top BLAST | |  | Top BLAST inf | |  | Highest cum | |  | Most freq | |  | Word-score | | Blannotator |
| --- | --- | --- | --- | --- | --- | --- | --- | --- | --- | --- | --- | --- | --- | --- | --- | --- | --- |
| ID% | COV% | Hits | All | GO |  | All | GO |  | All | GO |  | All | GO |  | All | GO |
| 0 | 0 | 3021 | 0.25 | 0.09 |  | 0.13 | 0.00 |  | 0.17 | 0.07 |  | 0.17 | 0.06 |  | 0.36 | 0.12 | 0.46 |
| 0 | 20 | 3020 | 0.24 | 0.07 |  | 0.13 | -0.07 |  | 0.18 | 0.06 |  | 0.16 | 0.06 |  | 0.36 | 0.11 | 0.46 |
| 0 | 40 | 3017 | 0.23 | 0.07 |  | 0.12 | 0.00 |  | 0.17 | 0.05 |  | 0.16 | 0.05 |  | 0.35 | 0.11 | 0.45 |
| 0 | 60 | 3013 | 0.22 | 0.08 |  | 0.10 | -0.06 |  | 0.16 | 0.04 |  | 0.15 | 0.04 |  | 0.33 | 0.09 | 0.44 |
| 0 | 80 | 3001 | 0.19 | 0.07 |  | 0.07 | -0.06 |  | 0.14 | 0.07 |  | 0.12 | 0.05 |  | 0.31 | 0.12 | 0.43 |
| 0 | 100 | 2057 | 0.06 | 0.06 |  | -0.10 | -0.10 |  | 0.03 | 0.06 |  | 0.03 | 0.06 |  | 0.12 | 0.09 | 0.28 |
| 20 | 0 | 3021 | 0.25 | 0.11 |  | 0.13 | 0.03 |  | 0.17 | 0.08 |  | 0.17 | 0.08 |  | 0.36 | 0.14 | 0.46 |
| 20 | 20 | 3020 | 0.24 | 0.10 |  | 0.13 | -0.02 |  | 0.18 | 0.07 |  | 0.16 | 0.07 |  | 0.36 | 0.12 | 0.46 |
| 20 | 40 | 3017 | 0.23 | 0.09 |  | 0.12 | 0.00 |  | 0.17 | 0.06 |  | 0.16 | 0.05 |  | 0.35 | 0.11 | 0.45 |
| 20 | 60 | 3013 | 0.22 | 0.13 |  | 0.10 | -0.01 |  | 0.16 | 0.09 |  | 0.15 | 0.08 |  | 0.33 | 0.14 | 0.44 |
| 20 | 80 | 3001 | 0.19 | 0.08 |  | 0.07 | -0.04 |  | 0.14 | 0.07 |  | 0.12 | 0.05 |  | 0.31 | 0.11 | 0.43 |
| 20 | 100 | 2057 | 0.06 | 0.05 |  | -0.10 | -0.10 |  | 0.03 | 0.05 |  | 0.03 | 0.05 |  | 0.12 | 0.07 | 0.28 |
| 40 | 0 | 3011 | 0.17 | 0.09 |  | 0.06 | -0.05 |  | 0.08 | 0.05 |  | 0.09 | 0.05 |  | 0.27 | 0.10 | 0.41 |
| 40 | 20 | 3009 | 0.16 | 0.07 |  | 0.06 | -0.04 |  | 0.09 | 0.04 |  | 0.10 | 0.05 |  | 0.27 | 0.10 | 0.41 |
| 40 | 40 | 3006 | 0.16 | 0.08 |  | 0.05 | -0.03 |  | 0.08 | 0.04 |  | 0.09 | 0.04 |  | 0.26 | 0.10 | 0.40 |
| 40 | 60 | 2999 | 0.15 | 0.08 |  | 0.05 | -0.06 |  | 0.08 | 0.03 |  | 0.09 | 0.04 |  | 0.25 | 0.09 | 0.39 |
| 40 | 80 | 2983 | 0.14 | 0.06 |  | 0.03 | -0.07 |  | 0.06 | 0.04 |  | 0.07 | 0.03 |  | 0.24 | 0.09 | 0.38 |
| 40 | 100 | 2026 | 0.06 | 0.04 |  | -0.10 | -0.10 |  | 0.01 | 0.03 |  | 0.02 | 0.04 |  | 0.11 | 0.06 | 0.27 |
| 60 | 0 | 2868 | 0.09 | 0.08 |  | -0.03 | -0.07 |  | 0.00 | 0.03 |  | 0.01 | 0.03 |  | 0.13 | 0.08 | 0.31 |
| 60 | 20 | 2865 | 0.09 | 0.07 |  | -0.03 | -0.06 |  | 0.00 | 0.02 |  | 0.01 | 0.02 |  | 0.13 | 0.07 | 0.31 |
| 60 | 40 | 2862 | 0.08 | 0.04 |  | -0.03 | -0.09 |  | 0.00 | 0.01 |  | 0.01 | 0.02 |  | 0.13 | 0.06 | 0.31 |
| 60 | 60 | 2854 | 0.08 | 0.07 |  | -0.03 | -0.09 |  | 0.00 | 0.02 |  | 0.01 | 0.03 |  | 0.13 | 0.08 | 0.31 |
| 60 | 80 | 2833 | 0.07 | 0.04 |  | -0.04 | -0.12 |  | 0.00 | 0.00 |  | 0.01 | 0.01 |  | 0.12 | 0.05 | 0.30 |
| 60 | 100 | 1908 | 0.05 | 0.06 |  | -0.11 | -0.11 |  | 0.00 | 0.03 |  | 0.00 | 0.04 |  | 0.08 | 0.06 | 0.24 |
| 80 | 0 | 2526 | 0.06 | 0.05 |  | -0.07 | -0.08 |  | -0.04 | 0.01 |  | -0.03 | 0.02 |  | 0.04 | 0.04 | 0.20 |
| 80 | 20 | 2526 | 0.06 | 0.06 |  | -0.07 | -0.07 |  | -0.04 | 0.02 |  | -0.03 | 0.02 |  | 0.04 | 0.05 | 0.20 |
| 80 | 40 | 2518 | 0.06 | 0.08 |  | -0.07 | -0.08 |  | -0.04 | 0.04 |  | -0.03 | 0.05 |  | 0.04 | 0.07 | 0.20 |
| 80 | 60 | 2510 | 0.05 | 0.06 |  | -0.07 | -0.10 |  | -0.04 | 0.02 |  | -0.03 | 0.02 |  | 0.04 | 0.05 | 0.20 |
| 80 | 80 | 2490 | 0.05 | 0.07 |  | -0.07 | -0.09 |  | -0.03 | 0.04 |  | -0.02 | 0.04 |  | 0.04 | 0.07 | 0.19 |
| 80 | 100 | 1717 | 0.04 | 0.06 |  | -0.12 | -0.11 |  | -0.04 | 0.02 |  | -0.04 | 0.02 |  | 0.03 | 0.05 | 0.17 |
| 100 | 0 | 664 | 0.04 | 0.07 |  | -0.23 | -0.20 |  | -0.03 | 0.01 |  | -0.02 | 0.01 |  | 0.00 | 0.01 | 0.11 |
| 100 | 20 | 663 | 0.04 | 0.04 |  | -0.23 | -0.22 |  | -0.03 | 0.00 |  | -0.02 | 0.00 |  | -0.01 | 0.01 | 0.11 |
| 100 | 40 | 660 | 0.04 | 0.04 |  | -0.18 | -0.17 |  | -0.03 | -0.01 |  | -0.02 | 0.00 |  | 0.00 | 0.01 | 0.11 |
| 100 | 60 | 657 | 0.04 | 0.06 |  | -0.18 | -0.16 |  | -0.03 | 0.00 |  | -0.01 | 0.01 |  | 0.00 | 0.01 | 0.11 |
| 100 | 80 | 650 | 0.04 | 0.04 |  | -0.28 | -0.28 |  | -0.02 | 0.01 |  | -0.01 | 0.01 |  | 0.00 | 0.02 | 0.10 |
| 100 | 100 | 330 | 0.02 | 0.02 |  | -0.24 | -0.23 |  | -0.05 | -0.03 |  | -0.03 | -0.01 |  | 0.00 | -0.02 | 0.10 |

**Table S6.** Standardized prediction quality of the tested automated protein function prediction methods. The table shows the mean and standard deviation of standardized mLDs of predictions, calculated over test proteins having at least one BLAST hit. Number of test proteins with at least one BLAST hit is shown in Hits-column. Standardization was done for each test sequence by comparing its quality values to those associated with its BLAST hits. Statistics were calculated several times after removal of BLAST hits with smaller than the given sequence identity (ID%) or alignment coverage (COV%) to the query sequence. DE were predicted based upon the most significant BLAST match (Top BLAST), the most significant BLAST match of which annotation did not had uninformative words (Top BLAST inf), the highest sum of bit scores (Highest cum), the frequency in the hit list (Most freq), the word importance based scoring method (Word-score) and BLANNOTATOR.

| ID% | COV% | Hits | Top BLAST | | | | |  | Top BLAST inf | | | | |  | Highest cum | | | | |  | Most freq | | | | |  | Word-score | | | | |  | BLANNOTATOR | |
| --- | --- | --- | --- | --- | --- | --- | --- | --- | --- | --- | --- | --- | --- | --- | --- | --- | --- | --- | --- | --- | --- | --- | --- | --- | --- | --- | --- | --- | --- | --- | --- | --- | --- | --- |
|  |  |  | ALL | |  | GO | |  | ALL | |  | GO | |  | ALL | |  | GO | |  | ALL | |  | GO | |  | ALL | |  | GO | |  |  |  |
|  |  |  | mean | sd |  | mean | sd |  | mean | sd |  | mean | sd |  | mean | sd |  | mean | sd |  | mean | sd |  | mean | sd |  | mean | sd |  | mean | sd |  | mean | sd |
| 0 | 0 | 3021 | 0.36 | 0.36 |  | 0.38 | 0.36 |  | 0.39 | 0.36 |  | 0.40 | 0.36 |  | 0.37 | 0.36 |  | 0.39 | 0.36 |  | 0.37 | 0.36 |  | 0.39 | 0.36 |  | 0.32 | 0.35 |  | 0.37 | 0.36 |  | 0.29 | 0.32 |
| 0 | 20 | 3020 | 0.36 | 0.36 |  | 0.39 | 0.36 |  | 0.39 | 0.36 |  | 0.41 | 0.36 |  | 0.36 | 0.36 |  | 0.39 | 0.36 |  | 0.37 | 0.36 |  | 0.39 | 0.36 |  | 0.32 | 0.35 |  | 0.38 | 0.36 |  | 0.28 | 0.32 |
| 0 | 40 | 3017 | 0.36 | 0.36 |  | 0.38 | 0.36 |  | 0.39 | 0.36 |  | 0.40 | 0.36 |  | 0.36 | 0.36 |  | 0.39 | 0.37 |  | 0.36 | 0.36 |  | 0.39 | 0.37 |  | 0.32 | 0.35 |  | 0.37 | 0.36 |  | 0.28 | 0.32 |
| 0 | 60 | 3013 | 0.36 | 0.36 |  | 0.38 | 0.36 |  | 0.39 | 0.36 |  | 0.40 | 0.36 |  | 0.36 | 0.36 |  | 0.39 | 0.36 |  | 0.36 | 0.36 |  | 0.39 | 0.36 |  | 0.32 | 0.35 |  | 0.37 | 0.36 |  | 0.28 | 0.32 |
| 0 | 80 | 3001 | 0.36 | 0.36 |  | 0.38 | 0.36 |  | 0.39 | 0.36 |  | 0.40 | 0.36 |  | 0.36 | 0.36 |  | 0.38 | 0.36 |  | 0.37 | 0.36 |  | 0.38 | 0.36 |  | 0.32 | 0.35 |  | 0.36 | 0.36 |  | 0.28 | 0.32 |
| 0 | 100 | 2057 | 0.36 | 0.35 |  | 0.36 | 0.35 |  | 0.39 | 0.36 |  | 0.39 | 0.37 |  | 0.37 | 0.36 |  | 0.36 | 0.35 |  | 0.37 | 0.35 |  | 0.36 | 0.35 |  | 0.34 | 0.35 |  | 0.35 | 0.35 |  | 0.29 | 0.32 |
| 20 | 0 | 3021 | 0.36 | 0.36 |  | 0.38 | 0.36 |  | 0.39 | 0.36 |  | 0.40 | 0.36 |  | 0.37 | 0.36 |  | 0.38 | 0.36 |  | 0.37 | 0.36 |  | 0.38 | 0.36 |  | 0.32 | 0.35 |  | 0.37 | 0.36 |  | 0.29 | 0.32 |
| 20 | 20 | 3020 | 0.36 | 0.36 |  | 0.38 | 0.36 |  | 0.39 | 0.36 |  | 0.40 | 0.36 |  | 0.36 | 0.36 |  | 0.38 | 0.36 |  | 0.37 | 0.36 |  | 0.39 | 0.36 |  | 0.32 | 0.35 |  | 0.37 | 0.36 |  | 0.28 | 0.32 |
| 20 | 40 | 3017 | 0.36 | 0.36 |  | 0.38 | 0.36 |  | 0.39 | 0.36 |  | 0.40 | 0.36 |  | 0.36 | 0.36 |  | 0.39 | 0.36 |  | 0.36 | 0.36 |  | 0.39 | 0.36 |  | 0.32 | 0.35 |  | 0.38 | 0.36 |  | 0.28 | 0.32 |
| 20 | 60 | 3013 | 0.36 | 0.36 |  | 0.37 | 0.36 |  | 0.39 | 0.36 |  | 0.40 | 0.37 |  | 0.36 | 0.36 |  | 0.38 | 0.36 |  | 0.36 | 0.36 |  | 0.38 | 0.36 |  | 0.32 | 0.35 |  | 0.36 | 0.36 |  | 0.28 | 0.32 |
| 20 | 80 | 3001 | 0.36 | 0.36 |  | 0.38 | 0.36 |  | 0.39 | 0.36 |  | 0.40 | 0.36 |  | 0.36 | 0.36 |  | 0.37 | 0.36 |  | 0.37 | 0.36 |  | 0.38 | 0.36 |  | 0.32 | 0.35 |  | 0.36 | 0.36 |  | 0.28 | 0.32 |
| 20 | 100 | 2057 | 0.36 | 0.35 |  | 0.36 | 0.35 |  | 0.39 | 0.36 |  | 0.39 | 0.37 |  | 0.37 | 0.36 |  | 0.36 | 0.35 |  | 0.37 | 0.35 |  | 0.36 | 0.35 |  | 0.34 | 0.35 |  | 0.36 | 0.35 |  | 0.29 | 0.32 |
| 40 | 0 | 3011 | 0.36 | 0.36 |  | 0.37 | 0.35 |  | 0.39 | 0.36 |  | 0.39 | 0.36 |  | 0.37 | 0.36 |  | 0.37 | 0.36 |  | 0.37 | 0.36 |  | 0.37 | 0.36 |  | 0.32 | 0.35 |  | 0.36 | 0.36 |  | 0.28 | 0.32 |
| 40 | 20 | 3009 | 0.36 | 0.36 |  | 0.37 | 0.36 |  | 0.39 | 0.36 |  | 0.40 | 0.36 |  | 0.37 | 0.36 |  | 0.37 | 0.36 |  | 0.36 | 0.36 |  | 0.37 | 0.36 |  | 0.32 | 0.35 |  | 0.36 | 0.36 |  | 0.28 | 0.32 |
| 40 | 40 | 3006 | 0.36 | 0.36 |  | 0.37 | 0.36 |  | 0.39 | 0.36 |  | 0.39 | 0.36 |  | 0.37 | 0.36 |  | 0.37 | 0.36 |  | 0.36 | 0.36 |  | 0.37 | 0.36 |  | 0.32 | 0.35 |  | 0.36 | 0.36 |  | 0.28 | 0.32 |
| 40 | 60 | 2999 | 0.36 | 0.36 |  | 0.37 | 0.36 |  | 0.39 | 0.36 |  | 0.39 | 0.36 |  | 0.37 | 0.36 |  | 0.37 | 0.36 |  | 0.36 | 0.36 |  | 0.37 | 0.36 |  | 0.32 | 0.35 |  | 0.35 | 0.36 |  | 0.28 | 0.32 |
| 40 | 80 | 2983 | 0.36 | 0.36 |  | 0.37 | 0.36 |  | 0.39 | 0.36 |  | 0.39 | 0.36 |  | 0.37 | 0.36 |  | 0.37 | 0.36 |  | 0.37 | 0.36 |  | 0.37 | 0.36 |  | 0.32 | 0.35 |  | 0.35 | 0.36 |  | 0.28 | 0.32 |
| 40 | 100 | 2026 | 0.36 | 0.35 |  | 0.36 | 0.36 |  | 0.39 | 0.36 |  | 0.39 | 0.37 |  | 0.37 | 0.36 |  | 0.36 | 0.36 |  | 0.36 | 0.35 |  | 0.36 | 0.36 |  | 0.34 | 0.35 |  | 0.35 | 0.35 |  | 0.29 | 0.32 |
| 60 | 0 | 2868 | 0.35 | 0.36 |  | 0.35 | 0.35 |  | 0.39 | 0.36 |  | 0.38 | 0.37 |  | 0.37 | 0.36 |  | 0.36 | 0.36 |  | 0.37 | 0.36 |  | 0.36 | 0.36 |  | 0.33 | 0.36 |  | 0.35 | 0.36 |  | 0.28 | 0.32 |
| 60 | 20 | 2865 | 0.35 | 0.36 |  | 0.36 | 0.36 |  | 0.39 | 0.36 |  | 0.38 | 0.37 |  | 0.37 | 0.36 |  | 0.36 | 0.36 |  | 0.37 | 0.36 |  | 0.36 | 0.36 |  | 0.33 | 0.36 |  | 0.35 | 0.36 |  | 0.28 | 0.32 |
| 60 | 40 | 2862 | 0.35 | 0.36 |  | 0.36 | 0.36 |  | 0.39 | 0.36 |  | 0.39 | 0.37 |  | 0.37 | 0.36 |  | 0.37 | 0.36 |  | 0.37 | 0.36 |  | 0.36 | 0.36 |  | 0.34 | 0.36 |  | 0.35 | 0.36 |  | 0.28 | 0.32 |
| 60 | 60 | 2854 | 0.35 | 0.36 |  | 0.35 | 0.35 |  | 0.39 | 0.36 |  | 0.39 | 0.37 |  | 0.37 | 0.36 |  | 0.36 | 0.36 |  | 0.36 | 0.36 |  | 0.36 | 0.36 |  | 0.34 | 0.36 |  | 0.35 | 0.36 |  | 0.28 | 0.32 |
| 60 | 80 | 2833 | 0.35 | 0.36 |  | 0.36 | 0.36 |  | 0.39 | 0.36 |  | 0.39 | 0.37 |  | 0.37 | 0.36 |  | 0.37 | 0.36 |  | 0.36 | 0.36 |  | 0.36 | 0.36 |  | 0.34 | 0.36 |  | 0.35 | 0.36 |  | 0.28 | 0.32 |
| 60 | 100 | 1908 | 0.35 | 0.35 |  | 0.35 | 0.35 |  | 0.39 | 0.36 |  | 0.38 | 0.37 |  | 0.36 | 0.36 |  | 0.35 | 0.35 |  | 0.36 | 0.36 |  | 0.35 | 0.35 |  | 0.34 | 0.35 |  | 0.35 | 0.35 |  | 0.29 | 0.32 |
| 80 | 0 | 2526 | 0.36 | 0.36 |  | 0.36 | 0.36 |  | 0.40 | 0.37 |  | 0.40 | 0.37 |  | 0.38 | 0.36 |  | 0.36 | 0.36 |  | 0.37 | 0.36 |  | 0.36 | 0.36 |  | 0.36 | 0.36 |  | 0.36 | 0.36 |  | 0.31 | 0.33 |
| 80 | 20 | 2526 | 0.36 | 0.36 |  | 0.35 | 0.36 |  | 0.40 | 0.37 |  | 0.40 | 0.37 |  | 0.38 | 0.36 |  | 0.36 | 0.36 |  | 0.37 | 0.36 |  | 0.36 | 0.36 |  | 0.35 | 0.36 |  | 0.35 | 0.36 |  | 0.31 | 0.33 |
| 80 | 40 | 2518 | 0.35 | 0.36 |  | 0.35 | 0.36 |  | 0.40 | 0.37 |  | 0.39 | 0.37 |  | 0.38 | 0.36 |  | 0.35 | 0.36 |  | 0.37 | 0.36 |  | 0.35 | 0.36 |  | 0.35 | 0.36 |  | 0.35 | 0.36 |  | 0.30 | 0.33 |
| 80 | 60 | 2510 | 0.36 | 0.36 |  | 0.36 | 0.36 |  | 0.40 | 0.37 |  | 0.40 | 0.37 |  | 0.37 | 0.36 |  | 0.36 | 0.36 |  | 0.37 | 0.36 |  | 0.36 | 0.36 |  | 0.35 | 0.36 |  | 0.35 | 0.36 |  | 0.30 | 0.33 |
| 80 | 80 | 2490 | 0.36 | 0.36 |  | 0.35 | 0.36 |  | 0.40 | 0.37 |  | 0.40 | 0.38 |  | 0.37 | 0.36 |  | 0.35 | 0.36 |  | 0.37 | 0.36 |  | 0.35 | 0.36 |  | 0.35 | 0.36 |  | 0.35 | 0.36 |  | 0.31 | 0.33 |
| 80 | 100 | 1717 | 0.35 | 0.35 |  | 0.35 | 0.35 |  | 0.39 | 0.36 |  | 0.39 | 0.37 |  | 0.37 | 0.36 |  | 0.36 | 0.36 |  | 0.37 | 0.36 |  | 0.36 | 0.36 |  | 0.35 | 0.36 |  | 0.35 | 0.36 |  | 0.31 | 0.33 |
| 100 | 0 | 664 | 0.35 | 0.36 |  | 0.35 | 0.35 |  | 0.41 | 0.39 |  | 0.41 | 0.39 |  | 0.37 | 0.36 |  | 0.36 | 0.36 |  | 0.37 | 0.36 |  | 0.36 | 0.36 |  | 0.36 | 0.36 |  | 0.36 | 0.36 |  | 0.32 | 0.34 |
| 100 | 20 | 663 | 0.35 | 0.36 |  | 0.35 | 0.35 |  | 0.41 | 0.39 |  | 0.41 | 0.38 |  | 0.37 | 0.36 |  | 0.36 | 0.36 |  | 0.37 | 0.36 |  | 0.36 | 0.36 |  | 0.36 | 0.36 |  | 0.36 | 0.36 |  | 0.32 | 0.34 |
| 100 | 40 | 660 | 0.35 | 0.36 |  | 0.36 | 0.35 |  | 0.41 | 0.38 |  | 0.41 | 0.38 |  | 0.37 | 0.36 |  | 0.36 | 0.36 |  | 0.36 | 0.36 |  | 0.36 | 0.36 |  | 0.36 | 0.36 |  | 0.36 | 0.36 |  | 0.32 | 0.34 |
| 100 | 60 | 657 | 0.35 | 0.35 |  | 0.35 | 0.35 |  | 0.41 | 0.38 |  | 0.40 | 0.38 |  | 0.36 | 0.36 |  | 0.36 | 0.36 |  | 0.36 | 0.36 |  | 0.36 | 0.36 |  | 0.36 | 0.36 |  | 0.36 | 0.36 |  | 0.32 | 0.34 |
| 100 | 80 | 650 | 0.35 | 0.35 |  | 0.35 | 0.35 |  | 0.41 | 0.38 |  | 0.41 | 0.38 |  | 0.36 | 0.36 |  | 0.35 | 0.36 |  | 0.36 | 0.36 |  | 0.35 | 0.36 |  | 0.35 | 0.36 |  | 0.35 | 0.36 |  | 0.32 | 0.34 |
| 100 | 100 | 330 | 0.32 | 0.35 |  | 0.33 | 0.35 |  | 0.38 | 0.38 |  | 0.38 | 0.39 |  | 0.34 | 0.36 |  | 0.34 | 0.36 |  | 0.34 | 0.36 |  | 0.34 | 0.36 |  | 0.33 | 0.35 |  | 0.33 | 0.36 |  | 0.29 | 0.33 |

**Table S8.** Notations as in Table S5. Function prediction was done from data having circularly referenced annotations.

| ID% | COV% | Hits | Top BLAST | | | | |  | Top BLAST inf | | | | |  | Highest cum | | | | |  | Most freq | | | | |  | Word-score | | | | |  | BLANNOTATOR | |
| --- | --- | --- | --- | --- | --- | --- | --- | --- | --- | --- | --- | --- | --- | --- | --- | --- | --- | --- | --- | --- | --- | --- | --- | --- | --- | --- | --- | --- | --- | --- | --- | --- | --- | --- |
|  |  |  | ALL | |  | GO | |  | ALL | |  | GO | |  | ALL | |  | GO | |  | ALL | |  | GO | |  | ALL | |  | GO | |  |  |  |
|  |  |  | mean | sd |  | mean | sd |  | mean | sd |  | mean | sd |  | mean | sd |  | mean | sd |  | mean | sd |  | mean | sd |  | mean | sd |  | mean | sd |  | mean | sd |
| 0 | 0 | 2313 | 0.57 | 0.33 |  | 0.58 | 0.33 |  | 0.63 | 0.33 |  | 0.65 | 0.33 |  | 0.56 | 0.35 |  | 0.56 | 0.35 |  | 0.56 | 0.35 |  | 0.56 | 0.35 |  | 0.54 | 0.35 |  | 0.55 | 0.35 |  | 0.50 | 0.32 |
| 0 | 20 | 2307 | 0.57 | 0.33 |  | 0.58 | 0.33 |  | 0.63 | 0.33 |  | 0.65 | 0.33 |  | 0.56 | 0.35 |  | 0.56 | 0.35 |  | 0.56 | 0.35 |  | 0.56 | 0.35 |  | 0.54 | 0.35 |  | 0.55 | 0.35 |  | 0.50 | 0.32 |
| 0 | 40 | 2292 | 0.57 | 0.33 |  | 0.58 | 0.33 |  | 0.63 | 0.33 |  | 0.65 | 0.33 |  | 0.55 | 0.35 |  | 0.56 | 0.35 |  | 0.55 | 0.35 |  | 0.56 | 0.35 |  | 0.54 | 0.35 |  | 0.55 | 0.35 |  | 0.50 | 0.32 |
| 0 | 60 | 2271 | 0.57 | 0.33 |  | 0.58 | 0.33 |  | 0.63 | 0.33 |  | 0.65 | 0.33 |  | 0.55 | 0.35 |  | 0.56 | 0.35 |  | 0.55 | 0.35 |  | 0.56 | 0.35 |  | 0.54 | 0.35 |  | 0.55 | 0.35 |  | 0.50 | 0.32 |
| 0 | 80 | 2210 | 0.57 | 0.33 |  | 0.58 | 0.33 |  | 0.63 | 0.33 |  | 0.65 | 0.33 |  | 0.55 | 0.35 |  | 0.56 | 0.34 |  | 0.55 | 0.35 |  | 0.56 | 0.34 |  | 0.53 | 0.35 |  | 0.55 | 0.35 |  | 0.50 | 0.32 |
| 0 | 100 | 524 | 0.54 | 0.33 |  | 0.54 | 0.33 |  | 0.67 | 0.36 |  | 0.67 | 0.36 |  | 0.53 | 0.34 |  | 0.53 | 0.34 |  | 0.53 | 0.33 |  | 0.53 | 0.33 |  | 0.52 | 0.34 |  | 0.52 | 0.34 |  | 0.50 | 0.32 |
| 20 | 0 | 2313 | 0.57 | 0.33 |  | 0.58 | 0.33 |  | 0.63 | 0.33 |  | 0.66 | 0.33 |  | 0.56 | 0.35 |  | 0.57 | 0.35 |  | 0.56 | 0.35 |  | 0.57 | 0.35 |  | 0.54 | 0.35 |  | 0.55 | 0.35 |  | 0.50 | 0.32 |
| 20 | 20 | 2307 | 0.57 | 0.33 |  | 0.58 | 0.33 |  | 0.63 | 0.33 |  | 0.66 | 0.33 |  | 0.56 | 0.35 |  | 0.57 | 0.35 |  | 0.56 | 0.35 |  | 0.57 | 0.35 |  | 0.54 | 0.35 |  | 0.55 | 0.35 |  | 0.50 | 0.32 |
| 20 | 40 | 2292 | 0.57 | 0.33 |  | 0.58 | 0.33 |  | 0.63 | 0.33 |  | 0.65 | 0.33 |  | 0.55 | 0.35 |  | 0.56 | 0.35 |  | 0.55 | 0.35 |  | 0.56 | 0.35 |  | 0.54 | 0.35 |  | 0.55 | 0.35 |  | 0.50 | 0.32 |
| 20 | 60 | 2271 | 0.57 | 0.33 |  | 0.58 | 0.33 |  | 0.63 | 0.33 |  | 0.65 | 0.33 |  | 0.55 | 0.35 |  | 0.56 | 0.35 |  | 0.55 | 0.35 |  | 0.56 | 0.35 |  | 0.54 | 0.35 |  | 0.54 | 0.35 |  | 0.50 | 0.32 |
| 20 | 80 | 2210 | 0.57 | 0.33 |  | 0.58 | 0.33 |  | 0.63 | 0.33 |  | 0.65 | 0.33 |  | 0.55 | 0.35 |  | 0.56 | 0.34 |  | 0.55 | 0.35 |  | 0.55 | 0.34 |  | 0.53 | 0.35 |  | 0.55 | 0.35 |  | 0.50 | 0.32 |
| 20 | 100 | 524 | 0.54 | 0.33 |  | 0.54 | 0.33 |  | 0.67 | 0.36 |  | 0.67 | 0.36 |  | 0.53 | 0.34 |  | 0.53 | 0.34 |  | 0.53 | 0.33 |  | 0.53 | 0.33 |  | 0.52 | 0.34 |  | 0.52 | 0.34 |  | 0.50 | 0.32 |
| 40 | 0 | 2031 | 0.56 | 0.34 |  | 0.56 | 0.34 |  | 0.64 | 0.34 |  | 0.65 | 0.34 |  | 0.54 | 0.35 |  | 0.55 | 0.35 |  | 0.54 | 0.35 |  | 0.54 | 0.35 |  | 0.53 | 0.35 |  | 0.54 | 0.35 |  | 0.50 | 0.33 |
| 40 | 20 | 2022 | 0.56 | 0.34 |  | 0.56 | 0.34 |  | 0.64 | 0.34 |  | 0.65 | 0.34 |  | 0.54 | 0.35 |  | 0.55 | 0.35 |  | 0.54 | 0.35 |  | 0.54 | 0.35 |  | 0.52 | 0.35 |  | 0.53 | 0.35 |  | 0.50 | 0.33 |
| 40 | 40 | 1998 | 0.56 | 0.34 |  | 0.56 | 0.34 |  | 0.64 | 0.34 |  | 0.65 | 0.34 |  | 0.54 | 0.35 |  | 0.54 | 0.35 |  | 0.53 | 0.35 |  | 0.54 | 0.35 |  | 0.52 | 0.35 |  | 0.53 | 0.35 |  | 0.50 | 0.33 |
| 40 | 60 | 1955 | 0.55 | 0.34 |  | 0.55 | 0.34 |  | 0.64 | 0.35 |  | 0.65 | 0.35 |  | 0.53 | 0.35 |  | 0.54 | 0.35 |  | 0.53 | 0.35 |  | 0.53 | 0.35 |  | 0.52 | 0.35 |  | 0.53 | 0.35 |  | 0.49 | 0.33 |
| 40 | 80 | 1871 | 0.55 | 0.34 |  | 0.55 | 0.34 |  | 0.63 | 0.35 |  | 0.64 | 0.35 |  | 0.53 | 0.35 |  | 0.53 | 0.35 |  | 0.53 | 0.35 |  | 0.53 | 0.35 |  | 0.52 | 0.35 |  | 0.52 | 0.35 |  | 0.49 | 0.33 |
| 40 | 100 | 403 | 0.52 | 0.33 |  | 0.52 | 0.33 |  | 0.65 | 0.37 |  | 0.65 | 0.37 |  | 0.51 | 0.33 |  | 0.51 | 0.33 |  | 0.50 | 0.33 |  | 0.50 | 0.33 |  | 0.49 | 0.34 |  | 0.49 | 0.34 |  | 0.48 | 0.33 |

**Table S7.** Notations as in Table S5. Function prediction was done after removal of BLAST hits with sequence identity greater than 50% to the query sequence.

| ID% | COV% | Hits | Top BLAST | | | | |  | Top BLAST inf | | | | |  | Highest cum | | | | |  | Most freq | | | | |  | Word-score | | | | |  | BLANNOTATOR | |
| --- | --- | --- | --- | --- | --- | --- | --- | --- | --- | --- | --- | --- | --- | --- | --- | --- | --- | --- | --- | --- | --- | --- | --- | --- | --- | --- | --- | --- | --- | --- | --- | --- | --- | --- |
|  |  |  | ALL | |  | GO | |  | ALL | |  | GO | |  | ALL | |  | GO | |  | ALL | |  | GO | |  | ALL | |  | GO | |  |  |  |
|  |  |  | mean | sd |  | mean | sd |  | mean | sd |  | mean | sd |  | mean | sd |  | mean | sd |  | mean | sd |  | mean | sd |  | mean | sd |  | mean | sd |  | mean | sd |
| 0 | 0 | 2395 | 0.47 | 0.36 |  | 0.48 | 0.36 |  | 0.48 | 0.36 |  | 0.50 | 0.36 |  | 0.47 | 0.37 |  | 0.48 | 0.36 |  | 0.47 | 0.37 |  | 0.48 | 0.36 |  | 0.44 | 0.37 |  | 0.47 | 0.36 |  | 0.40 | 0.33 |
| 0 | 20 | 2391 | 0.47 | 0.36 |  | 0.49 | 0.36 |  | 0.48 | 0.36 |  | 0.50 | 0.36 |  | 0.47 | 0.37 |  | 0.49 | 0.36 |  | 0.47 | 0.37 |  | 0.49 | 0.36 |  | 0.44 | 0.37 |  | 0.48 | 0.36 |  | 0.40 | 0.33 |
| 0 | 40 | 2382 | 0.47 | 0.36 |  | 0.48 | 0.36 |  | 0.48 | 0.36 |  | 0.49 | 0.36 |  | 0.46 | 0.37 |  | 0.47 | 0.36 |  | 0.47 | 0.37 |  | 0.47 | 0.36 |  | 0.44 | 0.37 |  | 0.46 | 0.36 |  | 0.40 | 0.33 |
| 0 | 60 | 2365 | 0.47 | 0.36 |  | 0.48 | 0.36 |  | 0.48 | 0.36 |  | 0.49 | 0.36 |  | 0.46 | 0.36 |  | 0.47 | 0.36 |  | 0.47 | 0.37 |  | 0.47 | 0.36 |  | 0.43 | 0.36 |  | 0.45 | 0.36 |  | 0.40 | 0.33 |
| 0 | 80 | 2333 | 0.46 | 0.36 |  | 0.47 | 0.36 |  | 0.47 | 0.36 |  | 0.48 | 0.36 |  | 0.46 | 0.36 |  | 0.47 | 0.36 |  | 0.46 | 0.36 |  | 0.47 | 0.36 |  | 0.43 | 0.36 |  | 0.46 | 0.36 |  | 0.39 | 0.33 |
| 0 | 100 | 842 | 0.46 | 0.35 |  | 0.46 | 0.35 |  | 0.49 | 0.37 |  | 0.49 | 0.36 |  | 0.46 | 0.36 |  | 0.46 | 0.36 |  | 0.46 | 0.35 |  | 0.46 | 0.35 |  | 0.45 | 0.35 |  | 0.45 | 0.35 |  | 0.43 | 0.34 |
| 20 | 0 | 2395 | 0.47 | 0.36 |  | 0.48 | 0.36 |  | 0.48 | 0.36 |  | 0.50 | 0.36 |  | 0.47 | 0.37 |  | 0.48 | 0.36 |  | 0.47 | 0.37 |  | 0.48 | 0.36 |  | 0.44 | 0.37 |  | 0.47 | 0.36 |  | 0.40 | 0.33 |
| 20 | 20 | 2391 | 0.47 | 0.36 |  | 0.49 | 0.36 |  | 0.48 | 0.36 |  | 0.50 | 0.36 |  | 0.47 | 0.37 |  | 0.49 | 0.36 |  | 0.47 | 0.37 |  | 0.49 | 0.36 |  | 0.44 | 0.37 |  | 0.47 | 0.36 |  | 0.40 | 0.33 |
| 20 | 40 | 2382 | 0.47 | 0.36 |  | 0.49 | 0.36 |  | 0.48 | 0.36 |  | 0.50 | 0.36 |  | 0.46 | 0.37 |  | 0.48 | 0.36 |  | 0.47 | 0.37 |  | 0.48 | 0.36 |  | 0.44 | 0.37 |  | 0.47 | 0.36 |  | 0.40 | 0.33 |
| 20 | 60 | 2365 | 0.47 | 0.36 |  | 0.48 | 0.36 |  | 0.48 | 0.36 |  | 0.49 | 0.36 |  | 0.46 | 0.36 |  | 0.47 | 0.36 |  | 0.47 | 0.37 |  | 0.48 | 0.36 |  | 0.43 | 0.36 |  | 0.46 | 0.36 |  | 0.40 | 0.33 |
| 20 | 80 | 2333 | 0.46 | 0.36 |  | 0.47 | 0.36 |  | 0.47 | 0.36 |  | 0.49 | 0.36 |  | 0.46 | 0.36 |  | 0.47 | 0.36 |  | 0.46 | 0.36 |  | 0.47 | 0.36 |  | 0.43 | 0.36 |  | 0.46 | 0.36 |  | 0.39 | 0.33 |
| 20 | 100 | 842 | 0.46 | 0.35 |  | 0.46 | 0.35 |  | 0.49 | 0.37 |  | 0.49 | 0.37 |  | 0.46 | 0.36 |  | 0.46 | 0.36 |  | 0.46 | 0.35 |  | 0.46 | 0.35 |  | 0.45 | 0.35 |  | 0.45 | 0.35 |  | 0.43 | 0.34 |
| 40 | 0 | 2251 | 0.46 | 0.36 |  | 0.47 | 0.36 |  | 0.47 | 0.36 |  | 0.48 | 0.36 |  | 0.46 | 0.37 |  | 0.47 | 0.36 |  | 0.45 | 0.37 |  | 0.46 | 0.36 |  | 0.43 | 0.37 |  | 0.45 | 0.36 |  | 0.39 | 0.33 |
| 40 | 20 | 2244 | 0.46 | 0.36 |  | 0.46 | 0.36 |  | 0.47 | 0.36 |  | 0.48 | 0.36 |  | 0.46 | 0.37 |  | 0.46 | 0.36 |  | 0.45 | 0.36 |  | 0.46 | 0.36 |  | 0.43 | 0.36 |  | 0.45 | 0.36 |  | 0.39 | 0.34 |
| 40 | 40 | 2228 | 0.46 | 0.36 |  | 0.46 | 0.36 |  | 0.47 | 0.36 |  | 0.48 | 0.36 |  | 0.45 | 0.37 |  | 0.46 | 0.36 |  | 0.45 | 0.36 |  | 0.46 | 0.36 |  | 0.43 | 0.37 |  | 0.45 | 0.36 |  | 0.38 | 0.34 |
| 40 | 60 | 2200 | 0.45 | 0.36 |  | 0.46 | 0.36 |  | 0.47 | 0.36 |  | 0.47 | 0.36 |  | 0.45 | 0.36 |  | 0.46 | 0.36 |  | 0.44 | 0.36 |  | 0.45 | 0.36 |  | 0.42 | 0.36 |  | 0.45 | 0.36 |  | 0.38 | 0.34 |
| 40 | 80 | 2138 | 0.45 | 0.36 |  | 0.45 | 0.36 |  | 0.46 | 0.36 |  | 0.46 | 0.36 |  | 0.44 | 0.36 |  | 0.45 | 0.36 |  | 0.44 | 0.36 |  | 0.45 | 0.36 |  | 0.42 | 0.36 |  | 0.44 | 0.36 |  | 0.38 | 0.34 |
| 40 | 100 | 649 | 0.42 | 0.35 |  | 0.43 | 0.35 |  | 0.46 | 0.36 |  | 0.46 | 0.37 |  | 0.42 | 0.35 |  | 0.42 | 0.35 |  | 0.43 | 0.35 |  | 0.43 | 0.35 |  | 0.41 | 0.35 |  | 0.42 | 0.35 |  | 0.41 | 0.34 |

**Table S9.** Notations as in Table S5. Function prediction was done from data having circularly referenced annotations, but after removal of BLAST hits with sequence identity greater than 50% to the query sequence.

|  |  |  | Top BLAST | |  | Top BLAST inf | |  | Highest cum | |  | Most freq | |  | Word-score | | Blannotator |
| --- | --- | --- | --- | --- | --- | --- | --- | --- | --- | --- | --- | --- | --- | --- | --- | --- | --- |
| ID% | COV% | Hits | All | GO |  | All | GO |  | All | GO |  | All | GO |  | All | GO |
| 0 | 0 | 3021 | -0.06 | -0.06 |  | -0.54 | -0.64 |  | 0.18 | 0.14 |  | 0.14 | 0.12 |  | 0.23 | 0.15 | 0.43 |
| 0 | 20 | 3020 | -0.06 | -0.06 |  | -0.55 | -0.65 |  | 0.18 | 0.12 |  | 0.14 | 0.11 |  | 0.23 | 0.14 | 0.43 |
| 0 | 40 | 3017 | -0.06 | -0.06 |  | -0.56 | -0.66 |  | 0.18 | 0.11 |  | 0.14 | 0.11 |  | 0.22 | 0.14 | 0.42 |
| 0 | 60 | 3013 | -0.07 | -0.08 |  | -0.59 | -0.66 |  | 0.18 | 0.11 |  | 0.15 | 0.11 |  | 0.22 | 0.13 | 0.41 |
| 0 | 80 | 3001 | -0.09 | -0.10 |  | -0.62 | -0.69 |  | 0.18 | 0.10 |  | 0.15 | 0.10 |  | 0.21 | 0.13 | 0.40 |
| 0 | 100 | 2057 | -0.03 | -0.05 |  | -0.94 | -0.99 |  | 0.08 | 0.06 |  | 0.11 | 0.11 |  | 0.13 | 0.10 | 0.21 |
| 20 | 0 | 3021 | -0.06 | -0.07 |  | -0.54 | -0.64 |  | 0.18 | 0.13 |  | 0.14 | 0.12 |  | 0.23 | 0.14 | 0.43 |
| 20 | 20 | 3020 | -0.06 | -0.06 |  | -0.55 | -0.62 |  | 0.18 | 0.12 |  | 0.14 | 0.11 |  | 0.23 | 0.14 | 0.43 |
| 20 | 40 | 3017 | -0.06 | -0.05 |  | -0.56 | -0.63 |  | 0.18 | 0.12 |  | 0.14 | 0.12 |  | 0.22 | 0.14 | 0.42 |
| 20 | 60 | 3013 | -0.07 | -0.07 |  | -0.59 | -0.66 |  | 0.18 | 0.11 |  | 0.15 | 0.11 |  | 0.22 | 0.13 | 0.41 |
| 20 | 80 | 3001 | -0.09 | -0.08 |  | -0.62 | -0.70 |  | 0.18 | 0.11 |  | 0.15 | 0.11 |  | 0.21 | 0.13 | 0.40 |
| 20 | 100 | 2057 | -0.03 | -0.04 |  | -0.94 | -0.99 |  | 0.08 | 0.07 |  | 0.11 | 0.11 |  | 0.13 | 0.10 | 0.21 |
| 40 | 0 | 3011 | -0.08 | -0.10 |  | -0.66 | -0.75 |  | 0.18 | 0.12 |  | 0.17 | 0.12 |  | 0.21 | 0.14 | 0.38 |
| 40 | 20 | 3009 | -0.08 | -0.08 |  | -0.66 | -0.72 |  | 0.18 | 0.12 |  | 0.16 | 0.12 |  | 0.21 | 0.14 | 0.38 |
| 40 | 40 | 3006 | -0.08 | -0.09 |  | -0.67 | -0.77 |  | 0.18 | 0.13 |  | 0.17 | 0.12 |  | 0.21 | 0.14 | 0.38 |
| 40 | 60 | 2999 | -0.08 | -0.09 |  | -0.69 | -0.76 |  | 0.18 | 0.12 |  | 0.17 | 0.12 |  | 0.21 | 0.14 | 0.37 |
| 40 | 80 | 2983 | -0.09 | -0.09 |  | -0.71 | -0.76 |  | 0.17 | 0.11 |  | 0.17 | 0.12 |  | 0.20 | 0.13 | 0.36 |
| 40 | 100 | 2026 | -0.03 | -0.04 |  | -1.01 | -1.05 |  | 0.08 | 0.08 |  | 0.12 | 0.12 |  | 0.13 | 0.11 | 0.21 |
| 60 | 0 | 2868 | -0.05 | -0.05 |  | -1.01 | -1.08 |  | 0.12 | 0.10 |  | 0.12 | 0.11 |  | 0.14 | 0.12 | 0.24 |
| 60 | 20 | 2865 | -0.05 | -0.05 |  | -1.01 | -1.08 |  | 0.12 | 0.09 |  | 0.12 | 0.10 |  | 0.14 | 0.10 | 0.24 |
| 60 | 40 | 2862 | -0.05 | -0.05 |  | -1.02 | -1.07 |  | 0.12 | 0.08 |  | 0.13 | 0.09 |  | 0.14 | 0.10 | 0.24 |
| 60 | 60 | 2854 | -0.05 | -0.05 |  | -1.05 | -1.10 |  | 0.12 | 0.09 |  | 0.13 | 0.10 |  | 0.14 | 0.11 | 0.24 |
| 60 | 80 | 2833 | -0.05 | -0.05 |  | -1.04 | -1.10 |  | 0.11 | 0.09 |  | 0.13 | 0.10 |  | 0.13 | 0.11 | 0.24 |
| 60 | 100 | 1908 | 0.01 | 0.00 |  | -1.08 | -1.13 |  | 0.08 | 0.07 |  | 0.11 | 0.10 |  | 0.12 | 0.10 | 0.19 |
| 80 | 0 | 2526 | 0.02 | 0.01 |  | -1.16 | -1.21 |  | 0.07 | 0.07 |  | 0.07 | 0.08 |  | 0.07 | 0.07 | 0.19 |
| 80 | 20 | 2526 | 0.02 | 0.02 |  | -1.16 | -1.20 |  | 0.07 | 0.07 |  | 0.07 | 0.08 |  | 0.07 | 0.06 | 0.19 |
| 80 | 40 | 2518 | 0.02 | 0.01 |  | -1.17 | -1.21 |  | 0.07 | 0.07 |  | 0.08 | 0.08 |  | 0.07 | 0.07 | 0.18 |
| 80 | 60 | 2510 | 0.02 | 0.00 |  | -1.17 | -1.23 |  | 0.07 | 0.07 |  | 0.08 | 0.07 |  | 0.07 | 0.06 | 0.18 |
| 80 | 80 | 2490 | 0.02 | 0.01 |  | -1.18 | -1.23 |  | 0.07 | 0.07 |  | 0.07 | 0.08 |  | 0.07 | 0.07 | 0.18 |
| 80 | 100 | 1717 | 0.03 | 0.02 |  | -1.08 | -1.13 |  | 0.08 | 0.08 |  | 0.08 | 0.08 |  | 0.09 | 0.08 | 0.18 |
| 100 | 0 | 664 | 0.07 | 0.09 |  | -1.05 | -1.06 |  | 0.04 | 0.03 |  | 0.03 | 0.01 |  | 0.01 | 0.00 | 0.14 |
| 100 | 20 | 663 | 0.07 | 0.08 |  | -1.05 | -1.06 |  | 0.04 | 0.04 |  | 0.03 | 0.03 |  | 0.01 | 0.01 | 0.14 |
| 100 | 40 | 660 | 0.08 | 0.09 |  | -1.05 | -1.04 |  | 0.04 | 0.04 |  | 0.03 | 0.02 |  | 0.00 | 0.00 | 0.14 |
| 100 | 60 | 657 | 0.07 | 0.08 |  | -1.05 | -1.05 |  | 0.04 | 0.05 |  | 0.03 | 0.03 |  | 0.00 | 0.01 | 0.14 |
| 100 | 80 | 650 | 0.07 | 0.07 |  | -1.06 | -1.06 |  | 0.04 | 0.03 |  | 0.03 | 0.02 |  | 0.00 | 0.00 | 0.13 |
| 100 | 100 | 330 | 0.04 | 0.02 |  | -0.81 | -0.85 |  | 0.01 | 0.00 |  | 0.01 | 0.00 |  | 0.03 | 0.02 | 0.14 |

**Table S11.** Notations as in Table S6. Function prediction was done from data having circularly referenced annotations.

|  |  |  | Top BLAST | |  | Top BLAST inf | |  | Highest cum | |  | Most freq | |  | Word-score | | Blannotator |
| --- | --- | --- | --- | --- | --- | --- | --- | --- | --- | --- | --- | --- | --- | --- | --- | --- | --- |
| ID% | COV% | Hits | All | GO |  | All | GO |  | All | GO |  | All | GO |  | All | GO |
| 0 | 0 | 2395 | 0.10 | 0.03 |  | 0.08 | -0.01 |  | 0.10 | 0.04 |  | 0.07 | 0.04 |  | 0.20 | 0.10 | 0.36 |
| 0 | 20 | 2391 | 0.10 | 0.01 |  | 0.08 | -0.03 |  | 0.09 | 0.03 |  | 0.07 | 0.03 |  | 0.20 | 0.06 | 0.35 |
| 0 | 40 | 2382 | 0.09 | 0.02 |  | 0.07 | 0.00 |  | 0.10 | 0.06 |  | 0.08 | 0.07 |  | 0.19 | 0.11 | 0.35 |
| 0 | 60 | 2365 | 0.09 | 0.03 |  | 0.07 | -0.02 |  | 0.10 | 0.08 |  | 0.09 | 0.07 |  | 0.20 | 0.13 | 0.35 |
| 0 | 80 | 2333 | 0.08 | 0.03 |  | 0.06 | -0.01 |  | 0.08 | 0.04 |  | 0.07 | 0.03 |  | 0.18 | 0.07 | 0.34 |
| 0 | 100 | 842 | 0.05 | 0.04 |  | -0.04 | -0.05 |  | 0.05 | 0.05 |  | 0.02 | 0.03 |  | 0.08 | 0.07 | 0.14 |
| 20 | 0 | 2395 | 0.10 | 0.03 |  | 0.08 | -0.01 |  | 0.10 | 0.05 |  | 0.07 | 0.06 |  | 0.20 | 0.10 | 0.36 |
| 20 | 20 | 2391 | 0.10 | 0.01 |  | 0.08 | -0.04 |  | 0.09 | 0.02 |  | 0.07 | 0.03 |  | 0.20 | 0.06 | 0.35 |
| 20 | 40 | 2382 | 0.09 | 0.00 |  | 0.07 | -0.03 |  | 0.10 | 0.05 |  | 0.08 | 0.04 |  | 0.19 | 0.08 | 0.35 |
| 20 | 60 | 2365 | 0.09 | 0.03 |  | 0.07 | -0.01 |  | 0.10 | 0.04 |  | 0.09 | 0.04 |  | 0.20 | 0.08 | 0.35 |
| 20 | 80 | 2333 | 0.08 | 0.02 |  | 0.06 | -0.01 |  | 0.08 | 0.05 |  | 0.07 | 0.04 |  | 0.18 | 0.08 | 0.34 |
| 20 | 100 | 842 | 0.05 | 0.05 |  | -0.04 | -0.03 |  | 0.05 | 0.06 |  | 0.02 | 0.04 |  | 0.08 | 0.08 | 0.14 |
| 40 | 0 | 2251 | 0.05 | 0.00 |  | 0.01 | -0.04 |  | 0.05 | 0.02 |  | 0.07 | 0.03 |  | 0.14 | 0.06 | 0.32 |
| 40 | 20 | 2244 | 0.05 | 0.01 |  | 0.01 | -0.03 |  | 0.05 | 0.02 |  | 0.07 | 0.03 |  | 0.14 | 0.05 | 0.31 |
| 40 | 40 | 2228 | 0.03 | -0.01 |  | 0.00 | -0.04 |  | 0.06 | 0.01 |  | 0.07 | 0.03 |  | 0.14 | 0.05 | 0.30 |
| 40 | 60 | 2200 | 0.04 | -0.01 |  | 0.00 | -0.05 |  | 0.06 | 0.01 |  | 0.08 | 0.03 |  | 0.14 | 0.04 | 0.29 |
| 40 | 80 | 2138 | 0.05 | 0.04 |  | 0.02 | -0.01 |  | 0.06 | 0.03 |  | 0.07 | 0.04 |  | 0.14 | 0.06 | 0.28 |
| 40 | 100 | 649 | 0.06 | 0.05 |  | -0.06 | -0.06 |  | 0.03 | 0.04 |  | 0.00 | 0.01 |  | 0.07 | 0.07 | 0.10 |

**Table S10.** Notations as in Table S6. Function prediction was done after removal of BLAST hits with sequence identity greater than 50% to the query sequence.

|  |  |  | Top BLAST | |  | Top BLAST inf | |  | Highest cum | |  | Most freq | |  | Word-score | | Blannotator |
| --- | --- | --- | --- | --- | --- | --- | --- | --- | --- | --- | --- | --- | --- | --- | --- | --- | --- |
| ID% | COV% | Hits | All | GO |  | All | GO |  | All | GO |  | All | GO |  | All | GO |
| 0 | 0 | 2313 | 0.07 | 0.04 |  | -0.32 | -0.45 |  | 0.07 | 0.05 |  | 0.05 | 0.06 |  | 0.15 | 0.11 | 0.39 |
| 0 | 20 | 2307 | 0.07 | 0.04 |  | -0.32 | -0.45 |  | 0.06 | 0.05 |  | 0.05 | 0.06 |  | 0.15 | 0.12 | 0.39 |
| 0 | 40 | 2292 | 0.06 | 0.03 |  | -0.33 | -0.45 |  | 0.07 | 0.05 |  | 0.07 | 0.07 |  | 0.15 | 0.11 | 0.39 |
| 0 | 60 | 2271 | 0.06 | 0.04 |  | -0.35 | -0.47 |  | 0.08 | 0.03 |  | 0.08 | 0.06 |  | 0.14 | 0.10 | 0.38 |
| 0 | 80 | 2210 | 0.06 | 0.01 |  | -0.40 | -0.51 |  | 0.07 | 0.04 |  | 0.08 | 0.06 |  | 0.14 | 0.10 | 0.36 |
| 0 | 100 | 524 | -0.05 | -0.04 |  | -1.01 | -1.03 |  | 0.04 | 0.05 |  | 0.07 | 0.08 |  | 0.09 | 0.10 | 0.17 |
| 20 | 0 | 2313 | 0.07 | 0.03 |  | -0.32 | -0.46 |  | 0.07 | 0.04 |  | 0.05 | 0.05 |  | 0.15 | 0.10 | 0.39 |
| 20 | 20 | 2307 | 0.07 | 0.02 |  | -0.32 | -0.46 |  | 0.06 | 0.02 |  | 0.05 | 0.03 |  | 0.15 | 0.10 | 0.39 |
| 20 | 40 | 2292 | 0.06 | 0.02 |  | -0.33 | -0.45 |  | 0.07 | 0.05 |  | 0.07 | 0.07 |  | 0.15 | 0.11 | 0.39 |
| 20 | 60 | 2271 | 0.06 | 0.02 |  | -0.35 | -0.45 |  | 0.08 | 0.04 |  | 0.08 | 0.06 |  | 0.14 | 0.12 | 0.38 |
| 20 | 80 | 2210 | 0.06 | 0.00 |  | -0.40 | -0.51 |  | 0.07 | 0.04 |  | 0.08 | 0.07 |  | 0.14 | 0.10 | 0.36 |
| 20 | 100 | 524 | -0.05 | -0.04 |  | -1.01 | -1.03 |  | 0.04 | 0.05 |  | 0.07 | 0.08 |  | 0.09 | 0.10 | 0.17 |
| 40 | 0 | 2031 | 0.05 | 0.02 |  | -0.52 | -0.61 |  | 0.07 | 0.06 |  | 0.09 | 0.09 |  | 0.13 | 0.09 | 0.30 |
| 40 | 20 | 2022 | 0.05 | 0.02 |  | -0.52 | -0.61 |  | 0.07 | 0.06 |  | 0.09 | 0.09 |  | 0.13 | 0.10 | 0.30 |
| 40 | 40 | 1998 | 0.04 | 0.02 |  | -0.51 | -0.59 |  | 0.07 | 0.05 |  | 0.09 | 0.08 |  | 0.13 | 0.09 | 0.30 |
| 40 | 60 | 1955 | 0.04 | 0.03 |  | -0.54 | -0.60 |  | 0.07 | 0.04 |  | 0.09 | 0.07 |  | 0.13 | 0.09 | 0.29 |
| 40 | 80 | 1871 | 0.05 | 0.03 |  | -0.56 | -0.62 |  | 0.07 | 0.05 |  | 0.09 | 0.07 |  | 0.13 | 0.09 | 0.29 |
| 40 | 100 | 403 | -0.05 | -0.04 |  | -1.13 | -1.14 |  | 0.04 | 0.05 |  | 0.07 | 0.07 |  | 0.08 | 0.08 | 0.14 |

**Table S12.** Notations as in Table S6. Function prediction was done from data having circularly referenced annotations, but after removal of BLAST hits with sequence identity greater than 50% to the query sequence.
